# Chemical Profiling and *In Vivo* Evaluation of Sea Buckthorn-Derived Matrices in *Drosophila melanogaster* Under Varied Dietary Regimes

**DOI:** 10.3390/nu18050824

**Published:** 2026-03-03

**Authors:** Melinda Héjja, Róbert Nagy, György Tankó, Ferenc Ádám Lóga, Bence Pecsenye, Gábor Bancea, Zibuyile Mposula, Zoltán Cziáky, Tünde Pacza, Endre Máthé

**Affiliations:** 1 Doctoral School of Food Sciences, Faculty of Agricultural and Food Sciences and Environmental Management, University of Debrecen, Böszörményi Str. 138, H-4032 Debrecen, Hungary; pecsenye.bence@agr.unideb.hu (B.P.); bancea.gabor@agr.unideb.hu (G.B.); mposula.zibuyile@agr.unideb.hu (Z.M.); 2 Institute of Nutrition Science, Faculty of Agricultural and Food Sciences and Environmental Management, University of Debrecen, Böszörményi Str. 138, H-4032 Debrecen, Hungary; nagy.robert@agr.unideb.hu (R.N.); gyorgy_tanko@yahoo.com (G.T.); logafeca@gmail.com (F.Á.L.); pacza.tunde@unideb.hu (T.P.); 3 Agricultural and Molecular Research Institute, University of Nyíregyháza, Sóstói Str. 31, H-4400 Nyíregyháza, Hungary; cziaky.zoltan@nye.hu; 4 Department of Pharmaceutical Chemistry, Faculty of Pharmacy, “Vasile Goldis” Western University of Arad, L. Rebreanu Str. 86, RO-310414 Arad, Romania

**Keywords:** *Hippophae rhamnoides*, phytochemicals, viability, development

## Abstract

**Background**: Sea buckthorn (*Hippophae rhamnoides* L.), the superfood of the present era, is widely recognized for its high content of nutrients and bioactive compounds. However, dietary products and by-products derived from different parts of the fruit differ markedly in their biochemical composition, which may influence their nutritional and biological effects. *Drosophila melanogaster* represents a well-established *in vivo* model for studying the impact of dietary components on nutritional status, development, and viability under defined nutritional conditions. **Methods**: Four sea buckthorn-derived matrices—seed flour, seed oil, pulp, and fruit pomace powder—were analyzed for fatty acid, amino acid, polyphenol, and antioxidant contents. Their effects were evaluated in *D. melanogaster* under zero-nutrient, normal-nutrient, and high-sugar diets, assessing viability and developmental dynamics across various product types and concentrations. **Results**: Substantial compositional differences were observed between the samples. Seed flour and fruit pomace powder were rich in proteins, essential amino acids, polyphenols, flavonoids, and condensed tannins, whereas seed oil predominantly contained fatty acids with limited antioxidant capacity. Consistent with these compositional profiles, diet- and product-specific biological effects were observed. Under zero-nutrient conditions, high concentrations of fruit pomace powder (100 g/L) supported larval and adult viability and resulted in developmental patterns comparable to those observed under a normal-nutrient diet. Under normal-nutrient and high-sugar diets, the matrices modulated development and viability without apparent toxicity, with fruit pomace powder consistently showing the most favorable effects. **Conclusions**: The biological responses of *D. melanogaster* are closely linked to the biochemical composition of the matrices and the dietary context. Fruit pomace powder emerged as the most effective product, highlighting its potential as a functional dietary ingredient and a valuable source of nutrients and bioactive compounds.

## 1. Introduction

In the last two decades, big pharmaceutical companies have been struggling to produce novel medicines to tackle complex diseases such as cancer, Type 2 diabetes mellitus (T2DM), or cardiovascular diseases (CVD), while the ever-increasing rate of antibiotic resistance further complicates the development of new drugs [1,2,3,4,5]. Recently, natural products often described in traditional medicine are living their renaissance not only in everyday life but in the fields of clinical sciences as well. Together with emerging trends of “health-conscious”, plant-based diets and lifestyles, plants with tremendous biochemical activity could be used to treat chronic illnesses and disabilities [3,6].

Sea buckthorn (*Hippophae rhamnoides* L.) is a deciduous shrub belonging to the *Elaeagnaceae* family, commonly found and grown from Central Europe to Central and East Asia. Nowadays, it is considered a “superfood”, as almost every part of the plant is a rich source of health-promoting natural compounds, and suitable for human nutrition. It has been described in Traditional Chinese Medicine and Indian Ayurveda as a wonder plant since ancient times due to its numerous health-improving effects [7,8]. Its leaves and berries were used to treat skin problems, metabolic disorders, and cardiovascular diseases [9]. Several of these effects, such as alleviation of type 2 diabetes mellitus, hepatoprotective effects, inhibition of cell proliferation, inflammation, and oxidative stress, have been studied extensively in the last decade, highlighting its pharmaceutical and nutritional importance [10,11]. These health benefits are closely related to the rich nutritional profile of the berries, which contain several biochemically active compounds such as phytosterols, phenols, flavonoids, and provitamins (A, C, E). They are also a good source of essential polyunsaturated fatty acids such as omega-3 and omega-6 acids, which are recognized for their importance in neuroprotection, anti-inflammatory mechanisms, and cardiovascular protection. Furthermore, sea buckthorn (SB) contains a valuable polyunsaturated fatty acid, namely palmitoleic acid (omega-7), which has some relevance in regulating metabolic disorders in living organisms [7,10,12,13].

Among the numerous prevailing chronic issues, T2DM is one of the leading causes of death worldwide and is more frequent among the younger population as well. In several studies, SB and SB-related extracts were evaluated for their ability to alleviate diabetic symptoms. SB oil extracts were found to be effective in lowering blood glucose levels, increasing insulin sensitivity, and modulating pathways related to metabolism, such as P13K/Akt. Among the fatty acids, palmitoleic acid (omega-7) was suggested to possess strong anti-diabetic effects; however, the evidence is somewhat controversial [14,15,16]. In another study, SB polyphenols and flavonoids were shown to have blood glucose-regulating effects and increase insulin sensitivity, highlighting their importance in metabolism regulation [17].

The physiological effects described in the previous sections are closely related to the processing technology of the plant. Matrices derived from SB berries with different technologies, such as drying, pressing, or oil extraction, may differ greatly in their health-related effects and physical properties, depending on their composition and solubility of different compounds. [10,18,19,20]. This allows us to develop foodstuffs with specific properties targeted at specified groups. Murariu and colab. (2025) [21] successfully implemented sea buckthorn products, such as fruit powder, by-product powder, and juice, to further fortify chocolate products with bioactive constituents and minerals, mainly vitamin C and phosphorus. They also reported significant differences between the levels of fortification, depending on the sea buckthorn products utilized, influencing the associated physiological effects of these products [21,22]. As these effects are closely related to their inner composition and synergies between complex groups of substances, it is imperative to know how these matrices affect the metabolism and development of living organisms *in vivo*.

*Drosophila melanogaster* is a long-known and used model animal in nutrigenomic studies, due, in part, to its simplicity and similar genetic traits to its human counterparts [23]. This makes it an excellent model system to study the physiological effects and mechanisms of bioactive materials on several chronic issues *in vivo* in a dose-dependent manner. Although there are several animal studies focusing on SB and its derivatives, so far, there have only been a couple of studies targeting fruit flies as a model system against chronic disorders. Among them, one study focused on T2DM progression in *Drosophila melanogaster*, but without any clear conclusion [24].

In this study, we aimed at evaluating the initial compositions of four different SB matrices regarding their content of major nutrients and bioactive composition to identify the most prominent SB product for functional food development. Due to the prevalence of T2DM not only in Hungary but worldwide, we have also focused on using *Drosophila melanogaster* as a model animal to analyze the potential of SB in alleviating T2DM-related inflammation, thus providing a novel and alternative source of medical food for diabetic patients.

## 2. Materials and Methods

### 2.1. The Manufacturing Technology of Sea Buckthorn Matrices

**Figure 1:**
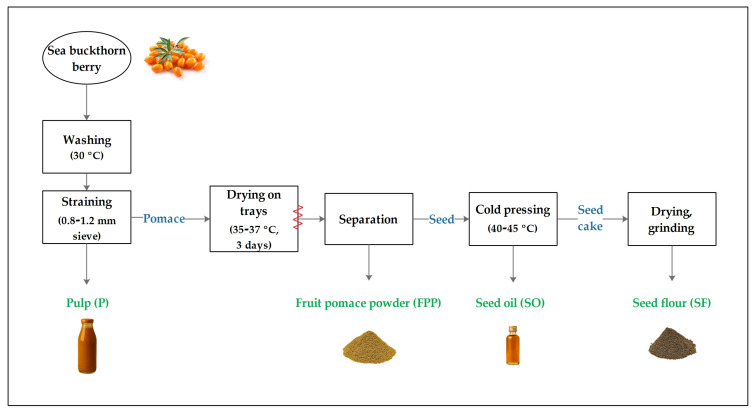
The processing procedure of the sea buckthorn berries.

Berries were collected from an organic plantation in Hajdú-Bihar County, Hungary (47°11′16.2″ N, 21°29′53.9″ E), in September 2024 and stored at 4 °C until processing. The processing procedure is illustrated in Figure 1. Berries were washed in water at 30 °C and strained on a 0.8–1.2 mm sieve to obtain pulp (P), while pomace remained as a by-product. The pomace was dried on trays at 35–37 °C for 3 days. After drying, seeds were separated from the pomace, and fruit pomace powder (FPP) was collected. Seeds were subsequently pressed to extract seed oil (SO). The residual seed cake was dried and ground to produce seed flour (SF).

### 2.2. Proximate Analysis of Sea Buckthorn Matrices

The chemical composition of the samples was analyzed in accordance with Hungarian standards, and since the measurements were performed in an accredited laboratory, the permissible analytical deviations are mentioned below.

Moisture content was measured based on the MSZ 4220:1980 Hungarian standard [25] by drying the samples at 103 °C to constant weight in an oven (Memmert B2171316, Memmert GmbH + Co. KG, Schwabach, Germany). Permissible analytical deviation was calculated as r = 0.593% + 0.0017·w, where w is the mean of the two replicate results, expressed in (m/m)%.

Protein content was quantified using the Kjeldahl method with a VELP UDK-149 distillation unit (VELP Scientifica, Usmate (MB), Italy) and a conversion factor of 6.38. The method is based on the MSZ 1385:1987 Hungarian standard [26]. Permissible analytical deviation was set to ±0.6 (m/m)%.

The total fat content was determined according to the MSZ 17617:1985 Hungarian standard [27] using a Soxhlet extractor (Tecator Soxtec System HT 1043 Extraction Unit, FOSS Tecator, Hilleroed, Denmark). Permissible analytical deviation was set to ±0.5 (m/m)%.

Dietary fiber content was determined by enzymatic hydrolysis according to Annex 2 of Directive No. 3-2-2008/1 of the Hungarian Food Book (Codex Alimentarius Hungaricus) [28], using dried, low-fat (<10%) or defatted samples. Permissible analytical deviation was ±5% (relative), expressed to the measured value.

The total sugar content was determined using the Luff–Schoorl method, in accordance with Section 7.3 of the MSZ 3625:1986 Hungarian standard [29]. The method was supplemented by an acid hydrolysis step, enabling the quantification of both the original reducing sugars and the non-reducing sugars released through inversion. Permissible analytical deviation was ±5% (relative), expressed relative to the measured value.

To determine the total carbohydrate content, the phenol–sulphuric acid method was used according to Lásztity and Törley (1987) ([30] Section 3.7.2.3) using a Whatman-type filter paper (Ahlstrom Munksjö, 150 mm, Grade 292, Ahlstrom-Munksjö, Helsinki, Finland). Permissible analytical deviation was ±10% (relative), expressed relative to the measured value.

### 2.3. Determination of Fatty Acid Profiles Using GC-FID

The fatty acid composition was determined using gas chromatography (GC). Analyses were performed on a Varian CP 3800 GC (Varian Inc., Palo Alto, CA, USA) equipped with a Restek Rt-2560 column (100 m × 0.25 mm ID, 0.20 µm film thickness, Restek Corp., Bellefonte, PA, USA).

The sample preparation is as follows: The extract obtained after the Soxhlet extraction (Section 2.2) was dissolved in 6 mL of *n*-hexane. An aliquot of 2 mL of the hexane solution was transferred to a headspace vial, and 4 mL of a NaOH:MeOH solution was added. The mixture was heated at 80 °C for 10 min in an oven to achieve saponification. After cooling, the reaction mixture was diluted with 5–10 mL of distilled water, and the unsaponifiable fraction was extracted twice with 2 mL of hexane each time; the combined hexane extracts (containing the waste material) were discarded. The remaining aqueous phase was acidified by adding 0.5 mL of 6 M H_2_SO_4_, and then fatty acids were extracted with 2 mL of hexane. The hexane extract was transferred to a new sealed vial, to which 2 mL of a 14% (*v*/*v*) boron trifluoride-methanol solution (BF_3_:MeOH) was added. The vial was heated at 80 °C for 30 min to form the corresponding fatty-acid methyl esters (FAMEs). Subsequently, 2 mL of saturated NaCl solution was added to promote phase separation; the upper hexane layer was withdrawn into a GC-injection vial pre-filled with anhydrous sodium sulfate. The sample was then analyzed by gas chromatography.

Sample injection was 1 µL, using an injector temperature of 250 °C and a split ratio of 1:50. Helium was used as the carrier gas at a constant flow rate of 1.9 mL/min. The oven temperature program was as follows: initial temperature of 150 °C held for 3 min, ramped at 2 °C/min to 240 °C, and held for 2 min. Detection was carried out using a flame ionization detector (FID) set at 250 °C. For the determination, Supelco 37 component FAME Mix was used as a standard (Sigma Aldrich, Darmstadt, Germany). The permissible analytical deviation was ±0.2% for results below 5 m/m% and ±1% for results above 5 m/m%. The lower limit of quantification was 0.01 (m/m)%.

### 2.4. Determination of Amino Acid Profiles

To determine amino acid profiles, protein hydrolysis was performed. The protein was transferred into a hydrolysis tube with a Teflon cap, mixed with 5 N HCl, and hydrolyzed at 105 °C for 5 h in an oven (Memmert UN55, Memmert GmbH + Co. KG, Büchenbach, Germany). After cooling, the hydrolysates were filtered through a regenerated cellulose filter (0.2 µm, Whatman 10463040 Spartan syringe filter, Cytiva, Little Chalfont, UK) and subjected to amino acid analysis.

Total amino acid analysis was carried out using an AAA500 amino acid analyzer (INGOS Ltd., Praha, Czechia) equipped with low-pressure ion-exchange chromatography and post-column derivatization with ninhydrin, followed by photometric detection at 210 and 254 nm. An amino acid standard mixture (INGOS Ltd., Praha, Czechia) was used as a reference. The recovery exceeded 95%. Permissible analytical deviation was ±10% (relative), expressed relative to the measured value.

Tryptophan was not determined by the acid hydrolysis-based amino acid analysis, as it is unstable under strong acidic conditions and undergoes degradation of the indole ring. Therefore, tryptophan content was determined separately using a colorimetric method based on sulfuric acid digestion and reaction with *p*-dimethylaminobenzaldehyde (VWR International, Leuven, Belgium), followed by spectrophotometric detection [31]. From the appropriately prepared (homogenized) sample, two parallel portions of 50 mg each were weighed into ground-glass test tubes. To one tube, 20 mL of 19 N sulfuric acid was added, while to the other tube, 20 mL of a 0.3% *p*-dimethylaminobenzaldehyde solution prepared in 19 N sulfuric acid was added. The tubes were kept in the dark for 16 h. Subsequently, 0.1 mL of a 0.045% sodium nitrite solution was added to the tube containing the color reagent. After a short reaction time, the solutions were filtered through a glass filter. The absorbance of the samples was measured at 590 nm. For quantification, a calibration curve was prepared using L-tryptophan (VWR International, Leuven, Belgium) standard solutions. Permissible analytical deviation was ±15% (relative), expressed relative to the measured value.

### 2.5. Analysis of Phytonutrient Profiles by UHPLC-ESI-MS/MS

The phytonutrient profile was determined according to Neamtu et al. (2020) [32]. The samples were analyzed using a UHPLC–Orbitrap MS system (Dionex Ultimate 3000RS, Thermo Fisher Scientific, Germering, Germany, with Thermo Q Exactive, Thermo Fisher Scientific, Bremen, Germany) on a C18 column in both positive and negative ESI modes. The separation used water–formic acid and methanol–formic acid eluents, a 200 μL/min flow rate, and a 70 min gradient program. Measurements had <5 ppm accuracy.

Phytochemicals were identified based on known standards (e.g., gallic acid, catechin, and quercetin derivatives) and also using Metlin, MassBank, and *m*/*z* Cloud databases and verified manually with Thermo Xcalibur 4.0 software (Thermo Fisher Scientific Inc.; Waltham, MA, USA)

### 2.6. Materials for Chemical Analysis

The chemicals used for the following analyses are summarized in Table S1 (See Supplementary Materials).

### 2.7. Determination of Antioxidant Activity

The antioxidant activities of the samples were evaluated using three standard assays: Ferric Reducing Antioxidant Power (FRAP), 2,2′-azino-bis(3-ethylbenzothiazoline-6-sulfonic acid) (ABTS) Radical Cation Decolorization Assay, and 2,2-diphenyl-1-picrylhydrazyl (DPPH) Free Radical Scavenging Assay. Sample preparation was identical for all assays: products were diluted at 1:10, vortexed for 30 min, and centrifuged at 13,400 rpm for 10 min. The only difference was the dilution solvent: distilled water for FRAP and methanol for ABTS and DPPH. All measurements were performed using a UV-Vis spectrophotometer (Ultrospec 2100 pro, Biochrom Ltd., Cambridge, UK).

#### 2.7.1. Ferric Reducing Antioxidant Power Assay (FRAP Assay)

The ferric reducing antioxidant power assay was performed following the method of Benzie and Strain (1996) [33], with minor modifications. The FRAP reagent was freshly prepared by mixing acetate buffer (300 mmol/L, pH 3.6), TPTZ solution (10 mmol/L), and FeCl_3_ (20 mmol/L) solution in a 10:1:1 ratio.

After that, 10 µL samples were added to the test tubes, followed by 2250 µL FRAP reagent. The mixtures were incubated for 5 min; absorbance was measured at 593 nm using the abovementioned spectrophotometer. Ascorbic acid (1 mg/mL) was used as the standard, and results were expressed as mg ascorbic acid equivalents (AAE) per mL.

#### 2.7.2. ABTS Radical Cation Decolorization Assay (ABTS Assay or TEAC Assay)

This assay followed the method of Re et al. (1999) [34], with minor modifications. The ABTS^+^· working solution was prepared by reacting 7 mM/L ABTS with 4.95 mM/L potassium persulfate in a 1:1 ratio and incubating the mixture in the dark for 12 h. Before use, the solution was diluted with distilled water (ten-fold dilution), and the pH was set to 7.7 with HCl. A 100 μL sample was mixed with 1 mL distilled water and 0.9 mL ABTS^+^ solution, and absorbance was measured at 734 nm after 20 min of incubation. A 1 mg/mL methanolic 6-hydroxy-2,5,7,8-tetramethylchroman-2-carboxylic acid (Trolox) solution was used as the calibration standard, and results were expressed as mg Trolox equivalents (TE) per mL (Trolox Equivalent Antioxidant Capacity—TEAC).

#### 2.7.3. DPPH Free Radical Scavenging Assay (DPPH Assay)

The DPPH radical scavenging assay was performed following Blois (1958) [35], with minor modifications. A 90 mg/L DPPH solution in methanol was prepared, and 100 μL of each sample was mixed with 1.4 mL of methanol and 1.5 mL of DPPH solution. Absorbance was measured at 517 nm after 30 min incubation. Trolox (1 mg/mL) was used as the standard, and results were expressed as mg Trolox equivalents (TE) per mL.

### 2.8. Determination of Total Phenolic and Flavonoid Content (TPC and TFC)

Sample preparation was identical for the two assays: products were diluted at 1:10 with methanolic distilled water (4:1), vortexed for 30 min, and centrifuged at 13,400 rpm for 10 min. Samples were measured using the Ultrospec 2100 pro UV/Vis spectrophotometer (Biochrom Ltd., Cambridge, UK).

#### 2.8.1. Total Phenolic Content (TPC)

TPC was determined with the Folin–Ciocâlteu method according to Singleton and Rossi (1965) [36], with minor modifications. First, 0.5 mL of each sample was mixed with 2.5 mL of 0.2 N Folin reagent, incubated for 5 min, and then 2 mL of 75 g/L Na_2_CO_3_ was added. Samples were kept in the dark for 2 h, and absorbance was measured at 760 nm. Gallic acid (100 mg/L) was used as the standard, and results were expressed as mg gallic acid equivalents (GAE) per mL.

#### 2.8.2. Total Flavonoid Content (TFC)

TFC was measured by the aluminum chloride colorimetric method [37], with minor modifications. First, 1 mL of each sample was mixed with distilled water (4 mL) and sequentially reacted with 5% NaNO_2_ (0.3 mL), 10% AlCl_3_ (0.3 mL), and 1 M NaOH (2 mL) solutions. Then, 2.4 mL of distilled water was added at the end. Samples were measured at 510 nm. Catechin (100 mg/L) was used as the standard, and results were expressed as mg catechin equivalents (CE) per mL.

### 2.9. Determination of Condensed Tannin Content (CTC)

Condensed tannin content (CTC) was determined using the vanillin–HCl assay, following Price et al. (1978) [38], with minor modifications by Hagerman (2002) [39]. Products were diluted at 1:10 with methanol, vortexed for 30 min, and centrifuged at 13,400 rpm for 10 min. Then, 1 mL of sample was mixed with the vanillin–HCl reagent (1% vanillin in methanol: 8% HCl in methanol, 1:1) and incubated at 30 °C for 20 min. Blanks were prepared for each sample using 4% HCl in methanol, instead of the vanillin–HCl reagent. Absorbance was measured at 500 nm, and catechin (0.3 mg/mL) was used as the standard. Results were expressed as mg catechin equivalents (CE) per mL.

### 2.10. The Drosophila melanogaster Strain Maintaining

*Drosophila melanogaster w^m4h^* (white mottled 4 h) strain was obtained from the Bloomington Drosophila Stock Center (BDSC) (Bloomington, IN, USA). The strain was maintained in bottles containing a culture medium composed of yeast paste (commercially available), sucrose (VWR International, Leuven, Belgium), wheat semolina (commercially available), agar (VWR International, Leuven, Belgium), and distilled water in a ratio of 7:5.135:3:1:100. For preparation, water was boiled in a Berzelius beaker on a heated magnetic stirrer, and yeast paste was mixed in until homogeneous. Sucrose and wheat semolina were then added and brought to a boil, after which agar was incorporated, and the mixture was brought to a boil again. After cooling the medium to 45 °C in a water bath, 1 g of Nipagin E (ethyl-4-hydroxybensoate; Thermo Fisher, Kandel, Germany) was added. The medium was poured into bottles and left to cool down. Flies were transferred to fresh media and incubated at 25 °C.

### 2.11. Preparation of the Drosophila melanogaster Culture Media for Experiments

The experiments were carried out on three different culture media, corresponding to three dietary conditions: zero-nutrient diet (0N-diet), normal-nutrient diet (NN-diet), and high-sugar diet (HS-diet). The composition of the media is detailed in Table 1.

For each medium, the first step involved bringing the water to a boil on a heated magnetic stirrer. For the zero-nutrient diet, agar was added and boiled again. For normal media and high-sugar media, commercially available yeast paste was added until a homogeneous mixture was obtained, and after that, sucrose and wheat semolina were also added, and the mixture was brought to a boil. After boiling, agar was added and boiled again.

The media were then cooled to 45 °C in a water bath, after which 1 g of Nipagin was thoroughly mixed in. The different sea buckthorn matrices were added directly to the media, and the prepared mixtures were poured into tubes. For normal and high-sugar media, five concentrations (50 g/L, 25 g/L, 12.5 g/L, 6.25 g/L, and 3.125 g/L) were prepared by serial twofold dilution. Each treatment was performed in triplicate, and control tubes containing no samples were also prepared. For the zero-nutrient diet, six concentrations (100 g/L, 50 g/L, 25 g/L, 12.5 g/L, 6.25 g/L, and 3.125 g/L) were prepared without control tubes. For liquid samples (pulp and seed oil), the concentrations were expressed in mL/L.

### 2.12. Experimental Design

To evaluate the effects of the different culture media, a randomized experimental design was implemented. The study involved three main treatment groups; each tested at 5–6 different concentrations (as described in Section 2.11). For each concentration, three independent replicates (vials) were established to ensure statistical reliability. Each replicate started with exactly 50 age-synchronized embryos to maintain a consistent density. The vials were kept under controlled environmental conditions (25 °C) throughout the observation period.

### 2.13. The Drosophila melanogaster Experiments

**Figure 2:**
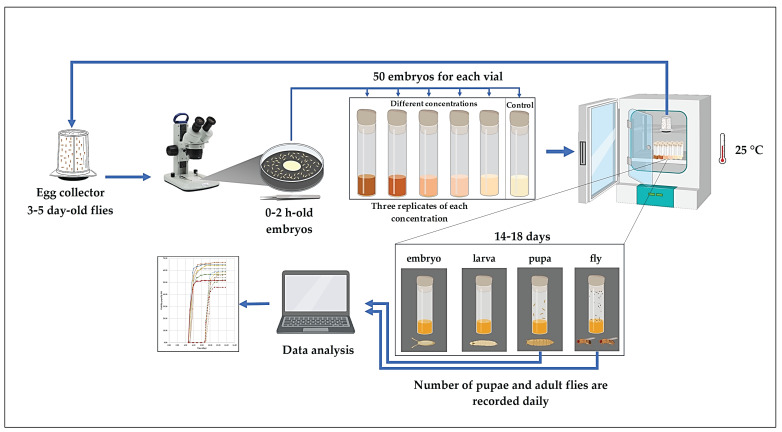
The scheme of the *Drosophila melanogaster* experiment.

The experiments were conducted according to the workflow shown in Figure 2. Approximately 500 five-day-old *Drosophila melanogaster* adults were transferred into an embryo collection cage placed above a Petri plate containing zero-nutrient medium supplemented with a yeast paste–water mixture. Embryo collection was performed continuously, with the plates being replaced every 2 h to obtain 0–2 h-old embryos. To ensure synchronization, the first two plates were discarded.

The embryos were carefully removed from the plates under a stereomicroscope using fine forceps and transferred into vials containing the corresponding zero-nutrient, normal-nutrient, or high-sugar media supplemented with the test samples.

The vials were incubated at 25 °C under constant humidity. The numbers of pupae and adult flies were recorded daily until no further pupae or adults emerged. The growth curve was expressed as proportions, calculated by dividing the daily number of newly formed pupae and emerged adult flies by the total number of individuals observed. Relative viability was calculated by dividing the total number of individuals formed at a given concentration by the total number formed in the control group.

### 2.14. Statistical Analysis

All measurements were conducted in triplicate, and results are expressed as the mean ± standard deviation (SD). Statistical analyses were performed using IBM SPSS Statistics version 26. Data were subjected to one-way analysis of variance (ANOVA), followed by Tukey’s HSD post hoc test, to identify significant differences between groups at *p* < 0.05.

## 3. Results

### 3.1. Comparative Evaluation of the Macronutrient Content of the Sea Buckthorn Matrices

The primary macronutrients—fats, proteins, and carbohydrates—serve as vital sources of energy and essential building blocks necessary for sustaining life. Achieving and maintaining optimal health and longevity depends on an appropriate balance of these macronutrients within the diet [40].

**Figure 3:**
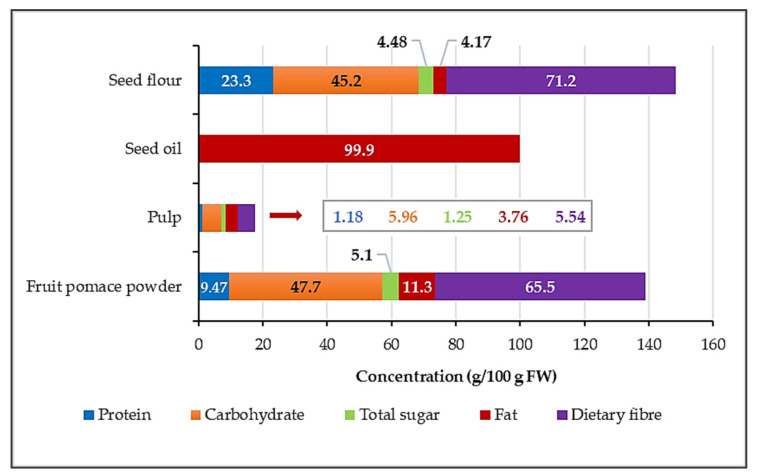
Macronutrient composition (g/100 g fresh weight) of the samples.

According to our analysis results (Figure 3), the dry matter content is 96.3% for fruit pomace powder, 13.2% for pulp, 99.9% for seed oil, and 91.2% for seed flour. The seed flour contains the highest amount of protein, making up 23.3% of its fresh weight, while the other products have significantly lower protein levels. The carbohydrate content of seed flour and fruit pomace powder is quite similar, accounting for nearly half of their fresh weight. As expected, oil consists entirely of fat (99.9 g/100 g FW), while FPP contains about 11.3 g/100 g FW. Regarding dietary fiber, seed flour and fruit pomace powder have the highest levels—71.2% and 65.5%, respectively—followed by pulp with 5.54%. There is a slight overlap between carbohydrate and dietary fiber concentration, as the method used to measure carbohydrates also measured some of the dietary fibers.

### 3.2. Comparative Evaluation of the Fatty Acid Profile of the Sea Buckthorn Matrices

Fats are essential for the body to function optimally, as they are the body’s main source of energy. They are categorized into saturated and unsaturated fatty acids, with certain types often referred to as “unhealthy” or “bad” fats [41]. Consequently, understanding the fatty acid composition of different foods is essential for making informed dietary choices and supporting overall health.

**Figure 4:**
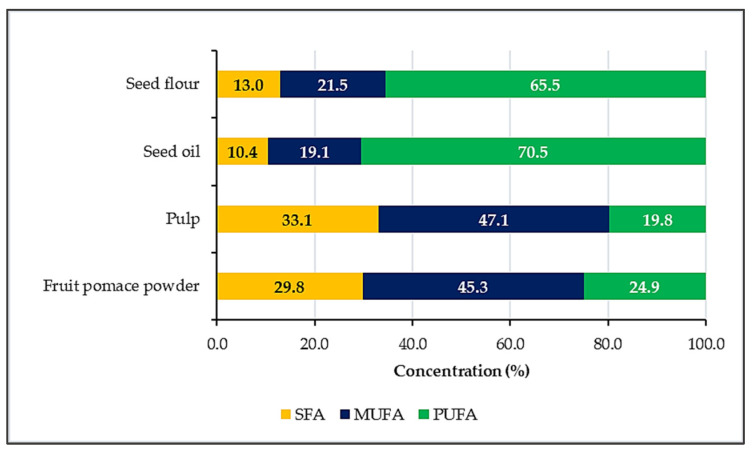
Proportion of saturated (SFA), monounsaturated (MUFA), and polyunsaturated (PUFA) fatty acids (% of total fat) in the sea buckthorn matrices.

When looking at the fat composition (Figure 4), monounsaturated fatty acids (MUFA) are dominant in pulp and fruit pomace powder, while in seed flour and oil, polyunsaturated fatty acids (PUFA) are more abundant. In pulp and fruit pomace powder, almost half of the total fatty acids are monounsaturated, though saturated fatty acids also make up a considerable portion (33.1% and 29.8%). Seed flour and oil stand out for their high PUFA content, accounting for 65.5% and 70.5% of their total fatty acids. These results highlight notable differences in fatty acid profiles between the products, with each showing a unique balance of saturated, monounsaturated, and polyunsaturated fats.

Based on the fatty acid profile data (Table 2), the seed oil contained the highest amount of fatty acids. The sample was dominated by unsaturated fatty acids, primarily cis-linoleic (36.77 g/100 g product), α-linolenic (33.20 g/100 g product), and oleic acids (18.40 g/100 g product), confirming its highly unsaturated nature. The product contained the highest amount of total saturated fatty acids, particularly palmitic acid (7.32 g/100 g product), followed by stearic acid (2.62 g/100 g product). This composition indicates that the seed oil is rich in both monounsaturated and polyunsaturated fatty acids, with a favorable ratio of unsaturated to saturated fats, making it potentially beneficial for dietary applications.

The seed flour showed a similar composition to seed oil but at reduced levels, characterized mainly by unsaturated fatty acids, reflecting the partial removal of oil during processing. Cis-linoleic acid (1.84 g/100 g product) was the predominant fatty acid, followed by oleic acid (0.88 g/100 g product) and α-linolenic acid (0.87 g/100 g product), while saturated fatty acids were minimal (palmitic acid 0.37 g/100 g product and stearic acid 0.14 g/100 g product).

The pulp sample showed considerably lower fatty acid levels overall, with minor contributions from palmitic, oleic, palmitoleic, and cis-linoleic acids. Among the saturated fatty acids, only palmitic acid was significant in an amount of 1.14 g/100 g of product. The MUFA and PUFA fraction was moderate, containing a lower amount of palmitoleic (0.87 g/100 g product), oleic (0.85 g/100 g product), cis-linoleic (0.60 g/100 g product), and α-linolenic acids (0.12 g/100 g product).

Fruit pomace powder contained the same fatty acids as pulp but in higher amounts. Palmitic acid (2.39 g/100 g product) was the main saturated fatty acid, while palmitoleic (2.44 g/100 g product) and oleic acids (2.09 g/100 g product) contributed substantially to the monounsaturated fraction. Polyunsaturated fatty acids were present mainly as α-linolenic acid (1.40 g/100 g product) and cis-linoleic acid (1.32 g/100 g product).

Overall, unsaturated fatty acids dominated in the seed oil, particularly cis-linoleic acid, α-linolenic acid, and oleic acid, while the fruit pomace powder was a rich source of both saturated and unsaturated fatty acids, such as palmitic, palmitoleic, oleic, cis-linoleic, and α-linolenic acid. Palmitic acid was the dominant fatty acid in the fruit pulp, while cis-linoleic acid was the most abundant in the seed flour. In terms of fat fraction, pulp and fruit pomace powder were richer in saturated and monounsaturated fatty acids, while seed oil and seed flour were dominated by unsaturated fatty acids, particularly cis-linoleic acid, α-linolenic acid, and oleic acid.

### 3.3. Comparative Evaluation of the Amino Acid Profile of the Sea Buckthorn Matrices

Amino acids are the major building blocks of proteins, and they play an important role in human metabolism, providing energy to the body and contributing to a healthy physical condition and the functioning of neurotransmitters [42].

Analyzing the amino acid profile data (Table 3), it can be concluded that seed flour is the richest source of amino acids, especially glutamine, asparagine, arginine, and proline, containing 5.84, 2.77, 1.90, and 1.68 g/100 g product, respectively. The essential amino acid profile is also relevant, containing more than 1 g/100 g product of leucine (1.33), tryptophan (1.24), and lysine (1.12). By analyzing the protein fraction, it can be concluded that glutamine is the most abundant, accounting for a quarter of the protein fraction. This is followed by asparagine (11.89), arginine (8.15), proline (7.21), leucine (5.71), serine (5.54), tryptophan (5.32), and alanine (5.02).

Fruit pomace powder is the second richest source of amino acids, containing relatively high concentrations of asparagine (1.89 g/100 g product), glutamine (1.53 g/100 g product), and proline (1.34 g/100 g product). The content of essential amino acids is lower than in seed flour, and the two EAAs with the highest concentrations are tryptophan (0.82 g/100 g product) and lysine (0.79 g/100 g product). In terms of protein fraction, the three abovementioned non-essential amino acids are the most abundant, followed by tryptophan (8.66), lysine (8.34), alanine (5.39), leucine (8.34), and serine (5.07).

The pulp contained amino acids only in low concentrations. Similar to the other products, asparagine, glutamine, and proline were the main amino acids, but in lower concentrations (0.1–0.5 g/100 g product) due to the higher water content. In the protein fraction, asparagine (33.9) and proline (17.8) were the main amino acids, followed by glutamine (9.32), lysine (6.78), and tryptophan (6.78). Since the seed oil did not contain protein, the amino acid profile was not determined.

Overall, asparagine, glutamine, and proline were the main amino acids in all three matrices. The main essential amino acid in the products was arginine, followed by leucine, lysine, and tryptophan.

### 3.4. Comprehensive Analysis of Phytonutrient Profile of Sea Buckthorn Matrices

The phytochemical profile of the matrices also includes several secondary metabolites that regulate the interaction between plants and the external environment. These non-nutrient compounds have disease-preventing properties and may have antioxidant, anti-inflammatory, and anticancer effects [43]. Table 4 summarizes the phytochemical profile of various sea buckthorn products. The results indicate that the pulp and fruit pomace powder exhibited the greatest diversity of flavonoids, including catechin, epicatechin, epigallocatechin, several quercetin derivatives, isorhamnetin, syringetin glucosides, rutinosides, and naringenin. FPP also contains serotonin and condensed tannins, such as procyanidin B. Seed flour was the third richest product, containing catechin, epicatechin, epigallocatechin, gallocatechin, isorhamnetin, kaempferol, and quercetin derivatives. It also contains serotonin and condensed tannins, such as prodelphinidin B isomer 1 and 2. Seed oil only contains malic acid, as other phytonutrients are absent.

### 3.5. Comparative Evaluation of Antioxidant Activity, Total Phenolic Content (TPC), Total Flavonoid Content (TFC), and Condensed Tannin Content (CTC) in Different Berry-Derived Matrices

Antioxidants are natural compounds that help protect the body’s cells from damage caused by harmful molecules called free radicals. These substances can slow down, regulate, or prevent oxidation processes in food or in the human body. Therefore, measuring the antioxidant activity of natural products is essential in studying their efficiency in preventing and treating diseases related to oxidative stress [44]. The most abundant antioxidants in our diet are polyphenols, which are compounds with one or more aromatic rings and hydroxyl groups. Clinical studies provide strong evidence that long-term consumption of polyphenol-rich diets can protect against the development of various chronic conditions, including neurodegenerative and cardiovascular diseases (CVDs), cancer, diabetes, inflammatory disorders, and infectious diseases [45].

**Figure 5:**
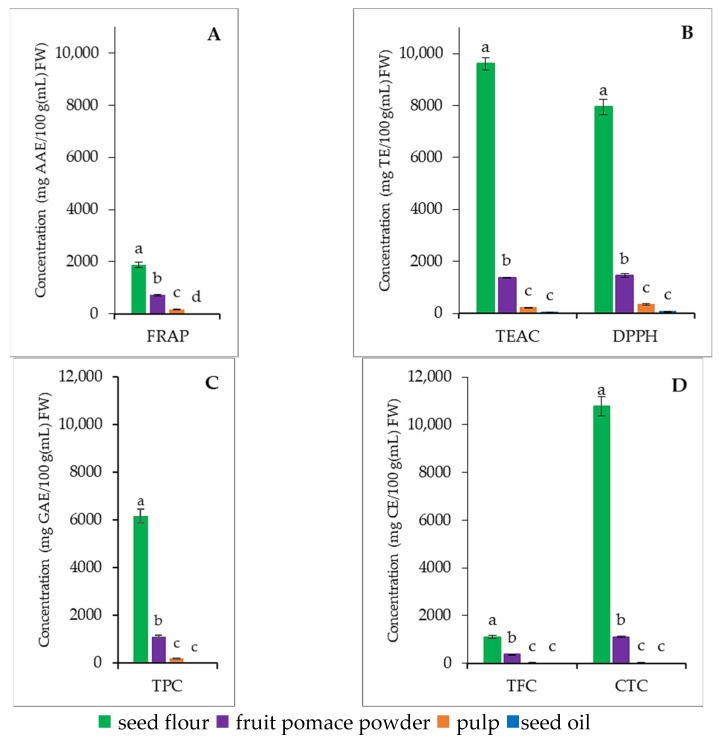
The antioxidant activity (**A**,**B**), total phenolic (**C**), flavonoid, and condensed tannin contents (**D**) of the different sea buckthorn matrices. FRAP, TEAC, and DPPH—total antioxidant capacity; TPC—total phenolic content; TFC—total flavonoid content; CTC—condensed tannin content. Values with different letters (a–d) within each method are statistically different at *p* < 0.05, according to Tukey’s test. Products are ranked by parameter value.

Based on our results (Figure 5A–D), seed flour (SF) exhibited the highest antioxidant activity across all analytical parameters, with DPPH, TEAC, and FRAP values of 7941.01 ± 289.47 mg TE/100 g FW, 9603.76 ± 231.21 mg TE/100 g FW, and 1872.78 ± 96.17 mg AAE/100 g FW, respectively. These elevated values are consistent with its high total phenolic (6152.99 ± 292.40 mg GAE/100 g FW), flavonoid (1108.80 ± 56.15 mg CE/100 g FW), and condensed tannin contents (10,772.43 ± 414.91 mg CE/100 g FW), indicating that its strong redox potential is likely driven by a rich polyphenolic profile.

In contrast, fruit pomace powder (FPP) and pulp (P) exhibited intermediate antioxidant potentials, showing statistically lower but still measurable activity in all assays, while seed oil (SO) demonstrated minimal or undetectable antioxidant capacity.

The statistical grouping (different superscript letters) confirms that these differences are highly significant (*p* < 0.05). Collectively, these results suggest that SF possesses superior radical scavenging and reducing power, which can be attributed to its elevated concentration of phenolic constituents, especially condensed tannins, underscoring its potential as a potent natural antioxidant source.

### 3.6. In Vivo Assessment of the Effects of Sea Buckthorn Matrices in the Drosophila melanogaster Model Organism

#### 3.6.1. Zero-Nutrient Diet (0N-Diet)

In a zero-nutrient diet, the culture medium lacks any external nutrients, and the tested products serve as the sole source of nutrition. The ability of embryos to develop, pupate, and hatch under these restrictive conditions serves as a biological indicator of the nutritional completeness of the matrices. This confirms that they provide the full spectrum of essential components required to support the complex developmental stages of a living organism, underscoring their potential as high-quality nutritional supplements.

**Figure 6:**
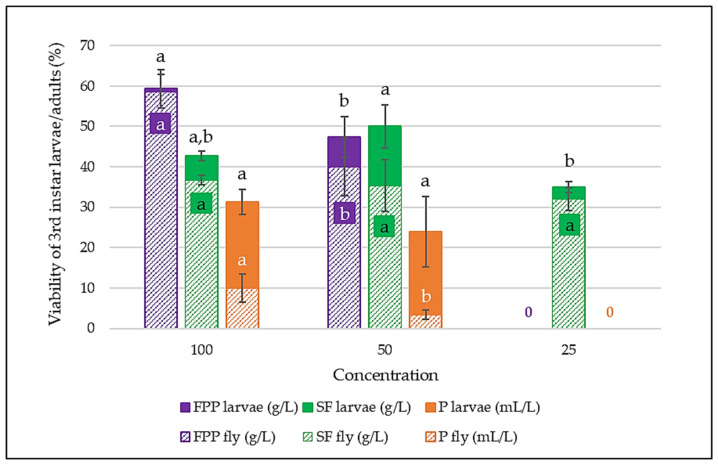
Viability of 3rd instar larvae and flies expressed as a percentage at different product concentrations on a zero-nutrient diet (SF—seed flour; P—pulp; FPP—fruit pomace powder). Values with different letters (a, b) within each product-stage are statistically different at *p* < 0.05, according to Tukey’s test. Matrices are ranked according to their performance at the highest concentration.

Our results (Figure 6) demonstrate that only the three highest concentrations (100, 50, and 25 g/L) of seed flour, pulp, and fruit pomace powder exerted a significant nutritive effect supporting larval development, whereas lower concentrations showed no observable impact. Fruit pomace powder yielded the highest third-instar larval viability (59.33%) at 100 g/L, with nearly all pupae successfully eclosing (98.88%). At 50 g/L, larval viability was 47.33%, and adult fly emergence reached 40%. Seed flour was the only treatment to exhibit nutritive effects at 25 g/L; however, adult fly viabilities across 100, 50, and 25 g/L (36.67%, 35.33%, and 32%, respectively) showed no significant differences (*p* > 0.05). Pulp powder displayed limited nutritive effects, restricted to 100 and 50 g/L, with larval viabilities below 30% in both cases and correspondingly weak adult emergence (10% and 3.33%). In contrast, the oil failed to support embryonic development at any tested concentration.

**Figure 7:**
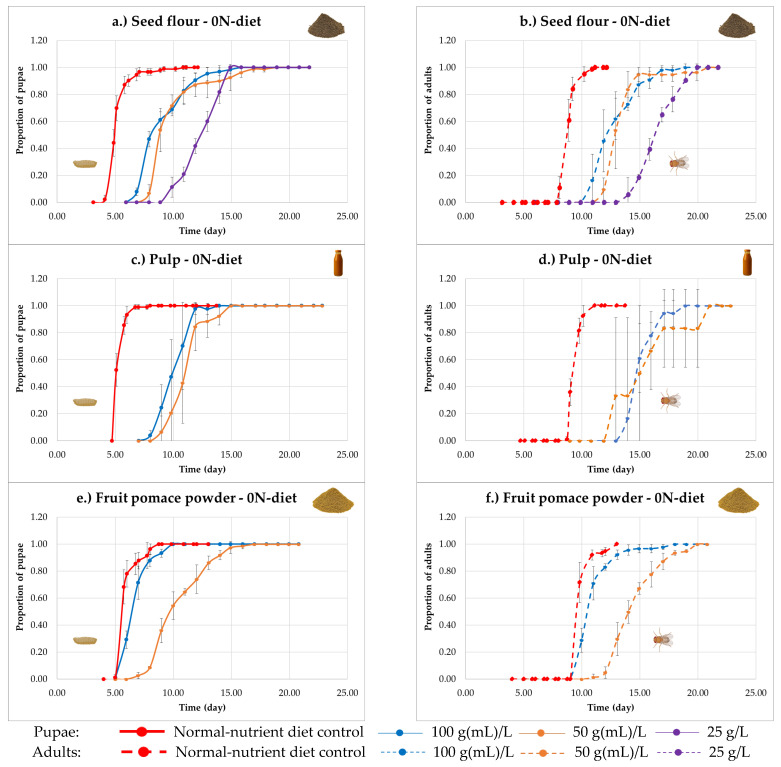
Time course of *Drosophila melanogaster* development under zero nutrient diet (0N-diet) supplemented with sea buckthorn seed flour ((**a**)—pupae, (**b**)—adults), pulp ((**c**)—pupae, (**d**)—adults) and fruit pomace powder ((**e**)—pupae, and (**f**)—adults). Proportions of pupae and adults are shown over time relative to the total individuals formed. The control curve comes from the normal-nutrient diet.

When developmental curves obtained on the zero-nutrient diet were compared to those of the normal-nutrient diet control (Figure 7), marked differences were observed between the tested products. In the case of seed flour and pulp, a pronounced developmental delay was detected at the highest concentration, reaching nearly three days relative to the normal-nutrient diet control. This delay showed a concentration-dependent pattern, with lower concentrations resulting in progressively greater delays.

In contrast, fruit pomace powder at the highest concentration (100 g/L) displayed a developmental curve closely resembling that of the normal-nutrient diet control, with comparable timing of pupation and adult emergence. At the lower concentration (50 g/L), however, a substantial developmental delay was observed, indicating a clear concentration-dependent response. Among the tested products, fruit pomace powder showed the smallest deviation from the control developmental pattern under zero-nutrient conditions.

#### 3.6.2. Normal-Nutrient Diet (NN-Diet) and High-Sugar Diet (HS-Diet)

The effects of sea buckthorn-derived matrices on the viability of *Drosophila melanogaster* were evaluated under normal-nutrient diet (NN-diet) and high-sugar diet (HS-diet) conditions. The high-sugar diet consists of five times the amount of sucrose (see Section 2.11), which can cause hyperglycemia, insulin resistance, and obesity in fruit flies [46,47]. Studies show that different plant extracts can suppress high glucose levels, increase emergence rate, and extend lifespan [48,49,50].

In our experiment, in both dietary regimes, viability followed a characteristic sigmoidal pattern, with the control groups displaying the expected developmental progression. In the case of a high-sugar diet, developmental delay can be observed, with pupal stage only beginning on the 6th or 7th day, whereas with a normal-nutrient diet, this process begins on the 4th or 5th day, but this phenomenon is a normal condition [51].

**Figure 8:**
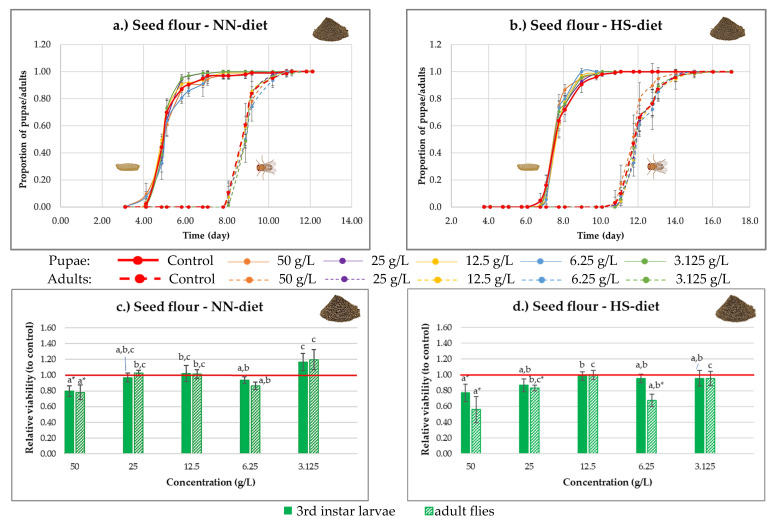
(**a**,**b**) Time course of *Drosophila melanogaster* development under normal-nutrient (NN-diet) and high-sugar (HS-diet) diets supplemented with sea buckthorn seed flour. Proportions of pupae and adults are shown over time relative to the total individuals formed. (**c**,**d**) Viability of 3rd instar larvae and adult flies relative to the control at different concentrations of sea buckthorn seed flour under NN-diet and HS-diet conditions. Values with different letters (a–c) within each stage are statistically different at *p* < 0.05, according to Tukey’s test. * statistically significant difference between the value and the control. The red horizontal line represents the control level (relative viability = 1.0), used as a reference for the normalization of results.

In the case of sea buckthorn seed flour, no effect on development was observed (Figure 8a,b). At all tested concentrations, the developmental curves closely followed those of the control group, indicating that this product neither promoted nor inhibited development.

Under NN-diet conditions (Figure 8c), the lowest concentration (3.125 g/L) showed a slight enhancement of survival (non-significant, *p* > 0.05), whereas the highest concentration (50 g/L) had a negative effect on viability compared to the control line, which is statistically significant (*p* < 0.05). Intermediate concentrations showed no significant deviation from the control.

Under HS-diet conditions (Figure 8d), almost all concentrations of seed flour negatively impacted the viability of third-instar larvae and adult flies, with the strongest effect observed at the highest concentration. These results suggest that while seed flour has minimal effects under normal dietary conditions, it may exacerbate the detrimental effects of a high-sugar diet on fly survival.

**Figure 9:**
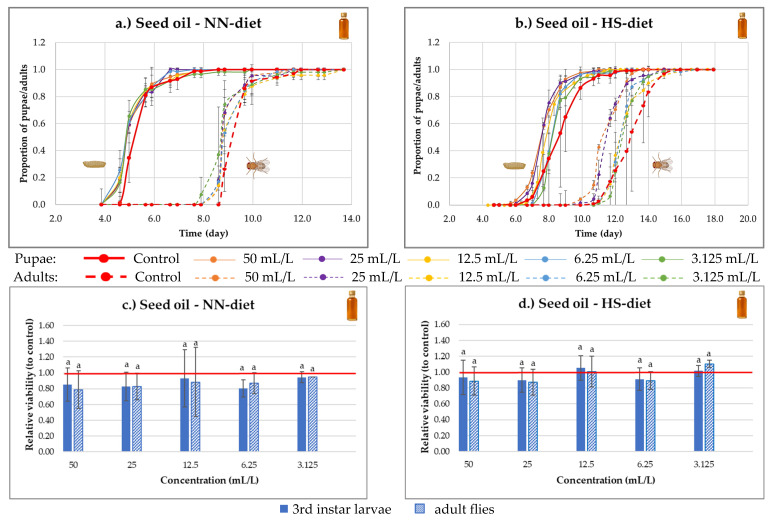
(**a**,**b**) Time course of *Drosophila melanogaster* development under normal-nutrient (NN-diet) and high-sugar (HS-diet) diets supplemented with sea buckthorn seed oil. Proportions of pupae and adults are shown over time relative to the total individuals formed. (**c**,**d**) Viability of 3rd instar larvae and adult flies relative to the control at different concentrations of sea buckthorn seed oil under NN-diet and HS-diet conditions. Values with the same letter within each stage are statistically indifferent at *p* < 0.05, according to Tukey’s test. The red horizontal line represents the control level (relative viability = 1.0), used as a reference for the normalization of results.

In the case of sea buckthorn seed oil, accelerated development was observed under both dietary conditions, with the effect being more pronounced under the HS-diet (Figure 9a,b). This acceleration was detectable across all tested concentrations, but it was most evident at the highest concentration (50 g/L). The enhanced developmental rate suggests that certain fatty acids in the seed oil may promote metabolic activity or modulate hormonal pathways involved in larval growth and metamorphosis. Possible mechanisms underlying this effect will be further discussed in the Section 4.

Under NN-diet conditions (Figure 9c), the lowest concentration (3.125 g/L) resulted in no difference compared to the control, whereas the other concentrations exerted a slight detrimental effect on viability (non-significant, *p* > 0.05), indicating a dose-dependent biphasic response, where low doses may be beneficial, while higher doses become harmful.

Under HS-diet conditions (Figure 9d), a similar trend was observed. The lowest concentration increased the viability of adult flies by approximately 11% (non-significant, *p* > 0.05), whereas higher concentrations either had no effect or reduced survival relative to the control. This pattern suggests that under metabolic stress induced by high sugar intake, low levels of seed oil may provide mild protective effects, possibly through lipid-balancing mechanisms.

**Figure 10:**
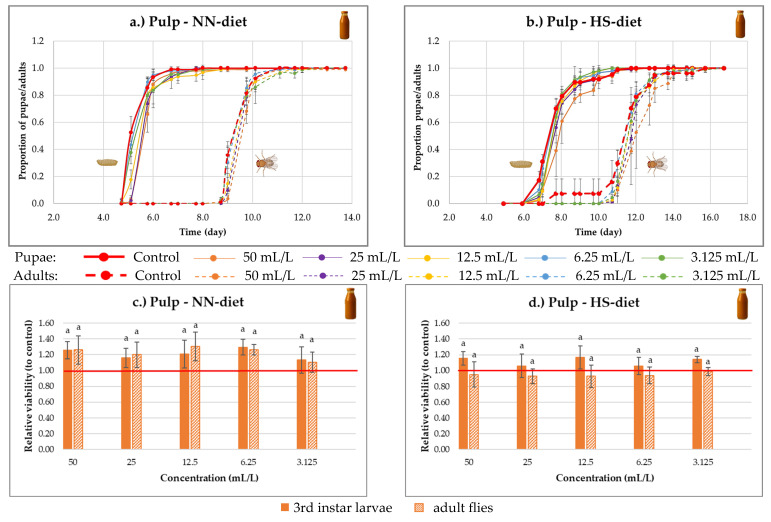
(**a**,**b**) Time course of *Drosophila melanogaster* development under normal-nutrient (NN-diet) and high-sugar (HS-diet) diets supplemented with sea buckthorn pulp. Proportions of pupae and adults are shown over time relative to the total individuals formed. (**c**,**d**) Viability of 3rd instar larvae and adult flies relative to the control at different concentrations of sea buckthorn pulp under NN-diet and HS-diet conditions. Values with the same letter within each stage are statistically indifferent at *p* < 0.05, according to Tukey’s test. The red horizontal line represents the control level (relative viability = 1.0), used as a reference for the normalization of results.

In the case of sea buckthorn pulp, a developmental delay develops under both diet conditions (Figure 10a,b), in which pupation and adult emergence occur a few hours later than in the control. This effect appears to be concentration-dependent: the greatest delay is observed at the highest concentration, while lower concentrations show progressively smaller differences, approaching control levels. Several possible explanations for this phenomenon will be addressed in the Section 4.

Relative viability analyses showed that under the NN-diet (Figure 10c), viability increased by 11–30% at different concentrations compared to the control (non-significant, *p* > 0.05). Under HS-diet conditions (Figure 10d), minor reductions were observed in adult fly viability, whereas the viability of third-instar larvae increased by up to 17% (non-significant, *p* > 0.05). These findings indicate that the pulp may exert mild nutritive effects under NN-diet conditions, whereas under the HS-diet, it only had a positive effect on the viability of third-instar larvae.

**Figure 11:**
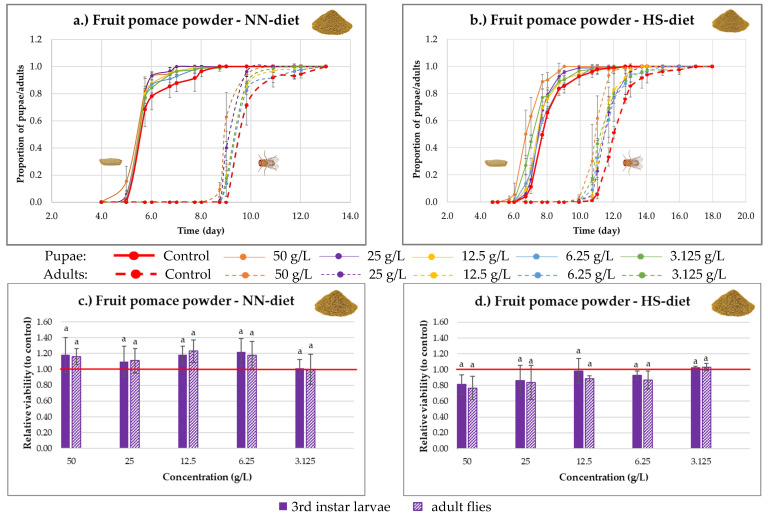
(**a**,**b**) Time course of *Drosophila melanogaster* development under normal-nutrient (NN-diet) and high-sugar (HS-diet) diets supplemented with sea buckthorn fruit pomace powder. Proportions of pupae and adults are shown over time relative to the total individuals formed. (**c**,**d**) Viability of 3rd instar larvae and adult flative to the control at different concentrations of sea buckthorn fruit pomace powder under NN-diet and HS-diet conditions. Values with the same letter within each stage are statistically indifferent at *p* < 0.05, according to Tukey’s test. The red horizontal line represents the control level (relative viability = 1.0), used as a reference for the normalization of results.

In the case of sea buckthorn fruit pomace powder, accelerated development was observed under both diet conditions, with a more pronounced effect under the HS-diet (Figure 11a,b). This phenomenon occurred at all concentrations but was most prominent at the highest concentration (50 g/L).

Under NN-diet conditions (Figure 11c), the relative viability of third-instar larvae and adults was higher compared to the control. Regarding concentrations, a similar trend was observed as in the case of the pulp, with the highest survival rates (up to 23%) occurring at 50, 12.5, and 6.25 g/L (non-significant, *p* > 0.05).

Under HS-diet conditions (Figure 11d), supplementation with fruit pomace powder had a negative effect on survival, which was concentration-dependent. The highest concentration showed the most negative effect (non-significant, *p* > 0.05), whereas the lowest concentration had no effect compared to the control.

## 4. Discussion

### 4.1. Compositional Characteristics of Sea Buckthorn Matrices

The four sea buckthorn products had clearly different biochemical profiles. The seed flour had a higher protein and fiber content than the other matrices, while the seed oil had the highest relative fatty acid content. Differences were also observed in the fatty acid composition, as seed flour and oil contained higher levels of polyunsaturated fatty acids, mainly linoleic acid and α-linolenic acid, while pulp and fruit pomace powder were richer in monounsaturated fatty acids, mainly palmitoleic acid and oleic acid. The amino acid profiles also differed between products, with seed flour and fruit pomace powder showing the greatest variety in terms of detectable amino acids, including essential amino acids. Significant differences were also observed in the content of antioxidants, polyphenols, flavonoids, and condensed tannins, with seed flour showing the highest levels, followed by fruit pomace powder. Overall, these compositional differences allowed grouping the products according to their dominant bioactive characteristics, which may provide a basis for understanding their differential biological effects in subsequent analyses. Similar variability in fatty acid, amino acid, and polyphenol contents between sea buckthorn products has been reported previously, supporting the heterogeneity of commercially available preparations.

The fatty acid profile of the sea buckthorn matrices was dominated by palmitoleic (0.65–23% of total fatty acids), oleic (18–23% of total fatty acids), linoleic (11–44% of total fatty acids), and α-linolenic acids (12–33% of total fatty acids), which were present in significant proportions in all products. The analysis of amino acids revealed the presence of several amino acids in the sea buckthorn matrices, with the greatest quantity observed in seed flour and fruit pomace powder. All the essential amino acids were detected, suggesting that sea buckthorn matrices may contain nutritionally relevant amino acid fractions. Significant differences in antioxidant capacity were observed between the sea buckthorn matrices. In all tests, seed flour consistently had the highest antioxidant potential (1872 mg AAE/100 g FW, 7941 mg TE/100 g FW, and 9603 mg TE/100 g FW), followed by fruit pomace powder, while sea buckthorn oil showed negligible antioxidant activity when evaluated using the FRAP, DPPH, and ABTS methods. It is noteworthy that the antioxidant values determined by the ABTS test were significantly higher than those obtained by the FRAP and DPPH methods, indicating significant differences between the methods. The predominance of higher ABTS-based values observed in the present study compared to FRAP and DPPH measurements is consistent with previous reports [52,53], suggesting that different antioxidant assays measure different fractions of the antioxidant potential of sea buckthorn products.

The fatty acid composition is consistent with previous reports describing sea buckthorn as a rich source of both monounsaturated and polyunsaturated fatty acids, like palmitoleic acid (28–35%) and oleic acid (17–24%) as the main monounsaturated components, along with significant amounts of essential linoleic acid (31% of total fatty acid in oil, 8% of total fatty acid in pomace) and α-linolenic acid (34% of total fatty acid in oil and 6% of total fatty acid in pomace) [54,55,56,57]. It is noteworthy that sea buckthorn is often characterized by a balanced ω-6:ω-3 fatty acid ratio close to 1:1 [55], which was observed in two of the four products examined in the present study (seed oil and fruit pomace powder). In contrast, a moderately shifted ω-6:ω-3 ratio was observed in seed flour (1:2), highlighting the variability in the composition of different sea buckthorn-derived matrices. Previous studies have shown the same amino acid spectrum in sea buckthorn juice as we did, but in varying amounts [7], pointing to the variability of amino acid composition depending on the type of product and its processing.

The significant variability in the antioxidant capacity of sea buckthorn products, including berries, processing by-products, and plant parts, has also been widely reported in the literature. These studies have shown extremely high antioxidant activity in sea buckthorn samples (9 mg TE/g in berries, 280 mmol TE/g DW in young shoots, and 120 mmol TE/g DW in press cake), although the reported values vary significantly depending on the test method used, the plant material, and the processing conditions [52,53,58,59]. The diversity of bioactive compounds of different sea buckthorn products and by-products was also represented in product development studies conducted by Murariu and colab (2025) [21]. They have reported highly variable data about sea buckthorn-fortified chocolates in different concentrations, and among them, chocolates with sea buckthorn fruit powders emerged as the highest source of polyphenols in the range of 0.48–0.84 mg GAE/g in different concentration, while they also showed strong antioxidant properties with values between 17 and 35 µmol TE/g via the CUPRAC method, showcasing the potential of sea buckthorn fruit in food industry and nutrition [21].

These pronounced compositional differences provided a suitable basis for examining whether distinct biochemical profiles translate into differential biological effects in *Drosophila melanogaster*.

### 4.2. Biological Responses of Drosophila melanogaster to Sea Buckthorn Matrices Across Different Diets

Three types of diets were used to study the different biological effects of sea buckthorn products: a zero-nutrient diet (0N-diet) to evaluate purely nutritive effects, a normal-nutrient diet (NN-diet) to identify potential toxic effects, and a high-sugar diet (HS-diet) to assess whether the matrices could alleviate sugar-induced stress.

Regarding these diets, two factors were evaluated: the developmental curve compared to the control and the relative viability.

In the nutrient-free diet, fruit pomace powder significantly improved the viability of both larvae and adult flies, indicating clear nutritional potential, followed by seed flour, while seed oil had no detectable nutritional effect. In the case of fruit pomace powder and seed flour, a considerable proportion of the pupae successfully completed metamorphosis and developed into adult flies. In contrast, pulp supplementation resulted in only a limited number of adult flies emerging, suggesting that although the early stages of development were partially supported, one or more factors necessary for the pupae to reach adulthood were lacking. The observed biological reactions were concentration-dependent, with fruit pomace powder and pulp only showing an effect at higher concentrations, while seed flour remained effective over a wider concentration range. These results suggest that sea buckthorn products differ significantly in their ability to support *Drosophila* development in the absence of external nutrients, highlighting qualitative differences in their nutritional completeness. Furthermore, the discrepancy observed between larval survival rates and adult emergence suggests that not all products are able to fully satisfy the nutritional requirements of later developmental stages. On the other hand, the observed developmental delays suggest that although certain sea buckthorn matrices may promote survival and partial development, they are not sufficient to completely replace the entire diet. Among the products, fruit pomace powder was the one that could partially compensate for the lack of a complete diet, but only if used in sufficiently high concentrations.

Under normal-nutrient dietary conditions, fruit pomace powder moderately altered both viability and developmental dynamics compared to the control. While overall survival was slightly higher than in the control, a slight positive shift in the developmental curve was observed, especially in the case of flies, suggesting a potential influence of the product on developmental processes. Seed oil showed a similar positive shift in the developmental curve. However, it had minimal negative effects on both larval and adult viability, which may reflect the impact of its high fat content. In contrast, seed flour did not adversely affect *Drosophila* development or viability compared to the control. Larval survival and adult emergence remained comparable to control values, and the developmental curve showed no marked delay or acceleration, indicating the absence of toxic effects. Pulp showed an interesting phenomenon: although it had a positive effect on viability, a developmental delay was observed, suggesting slower progression through the larval and pupal stages.

The high-sugar diet used in this study is a well-established *Drosophila* model for studying metabolic stress and insulin resistance, often used to model key features of type 2 diabetes. Under these conditions, fruit pomace powder and seed oil influenced developmental dynamics in a concentration-dependent manner, showing a slight positive shift in the developmental curve. However, overall viability was slightly lower than in the control, though not significantly. Seed flour did not alter the developmental curve, but it had a slight negative effect on viability, indicating that it could not alleviate sugar-induced stress. In contrast, pulp showed a positive effect on viability, but a developmental delay was observed, showing slower progression through larval and pupal stages.

These results show that the effect of sea buckthorn products on *Drosophila* development depends on both the product and the diet, suggesting that it may be related to their biochemical composition.

### 4.3. Composition-Dependent Differences in Drosophila Responses

The differences observed in the development and viability of *Drosophila* between sea buckthorn products may be related to their different biochemical compositions. Fruit pomace powder, which is characterized by a balanced level of saturated, mono- and polyunsaturated fatty acids, essential amino acids, carbohydrates, and phenolic compounds, has consistently had a positive effect on both viability and developmental progress, especially under nutrient-deficient conditions. This suggests that the combination of protein, fiber, and bioactive components may provide more complete nutritional support.

Pulp, which is also rich in phenolic compounds, fatty acids, and some amino acids, had more variable effects. While viability was sometimes enhanced, developmental delays were observed, indicating that the presence of certain nutrients may be insufficient or imbalanced to fully support the developmental process.

Seed flour, despite containing a variety of bioactive components, had almost no effect on survival and growth curves under normal and high-sugar diets. However, it had a good nutritional effect on a nutrient-free diet, suggesting that it contains all the nutrients necessary for fly metamorphosis.

Seed oil, rich in fatty acids but low in protein and antioxidants, showed limited positive effects on viability, with minor reductions in some cases. The observed developmental acceleration may be partly supported by the available energy from lipids, yet the low content of other essential nutrients likely limits overall developmental success.

These results suggest that differences in *Drosophila* responses likely reflect differences in the nutrient and bioactive compound profiles of the products. This warrants investigation of the mechanistic links between specific biochemical components and the observed developmental and viability effects in the next phase.

### 4.4. Potential Contribution of Sea Buckthorn Compounds to Drosophila melanogaster Responses

To better understand the observed effects on *Drosophila* development and viability, we examined how specific biochemical components of the sea buckthorn products may contribute to these responses.

Saturated and unsaturated fatty acids are essential components of biological systems, functioning primarily as energy sources and structural elements of cell membranes. In *Drosophila melanogaster*, fatty acids are required for normal growth, membrane integrity, and hormone-regulated developmental processes [60]. However, their biological effects strongly depend on both their concentration and relative composition.

Sea buckthorn matrices contained mainly C16–C20 fatty acids, with palmitic, palmitoleic, oleic, linoleic, and α-linolenic acids being the most abundant. Palmitic acid is the most common fatty acid in the human body [61] and has long been associated with adverse metabolic effects, including inflammation and dyslipidemia, particularly when present at high levels or under imbalanced dietary conditions [62]. Nevertheless, palmitic acid is also essential for normal cellular function, contributing to membrane structure and protein palmitoylation, and has been reported to exhibit antiviral, anti-inflammatory, and regulatory roles in lipid metabolism [61,63,64]. Short-term, low-dose exposure has even been shown to improve mitochondrial function and reduce oxidative stress in cell models [65]. These findings suggest that palmitic acid may exert beneficial or detrimental effects depending on dose and dietary context, which may explain the limited positive or slightly negative viability effects observed for seed oil in the present study.

Monounsaturated and polyunsaturated fatty acids (MUFAs and PUFAs) play an important role in maintaining membrane flexibility and regulating metabolic and antioxidant signaling pathways [66,67,68,69]. In *Drosophila*, such properties may influence developmental timing and stress adaptation by supporting efficient energy metabolism and cellular homeostasis. The relatively high C18 fatty acid content of fruit pomace powder and seed oil can be linked to positive changes in developmental dynamics observed under certain nutritional conditions [60].

Among PUFAs, ω-3 and ω-6 fatty acids are particularly important due to their opposing roles in inflammatory regulation. α-Linolenic acid (ω-3) is an essential fatty acid that cannot be synthesized endogenously and must be obtained from the diet [70]. It also serves as a precursor for longer-chain PUFAs, such as EPA and DHA, which are involved in the regulation of oxidative stress and inflammatory responses [66,67,71,72,73]. In contrast, high intake of ω-6 fatty acids is closely related to several cardiovascular diseases, obesity, and pro-inflammatory pathways [74]. Sea buckthorn has been reported to possess a favorable ω-6:ω-3 ratio close to 1:1 [55], and seed oil and fruit pomace powder examined in this study also followed this balanced profile. In *Drosophila melanogaster*, this ratio may support improved development and increased stress tolerance, particularly under metabolically challenging conditions such as a high-sugar diet.

Sea buckthorn also contains palmitoleic acid (omega-7), which is very rare in nature, making this berry one of its primary sources. This fatty acid and oleic acid—although not essential fatty acids—have gained increasing attention due to their reported roles in metabolic regulation, insulin sensitivity, and neuroprotective effects in animal and cellular models [16,75,76,77]. The presence of these fatty acids in sea buckthorn products may therefore further contribute to the observed biological responses in *Drosophila melanogaster*.

Although fatty acids are frequently discussed in the context of human health, including cardiovascular and metabolic diseases [74,78,79,80,81,82], their core biological functions are conserved across species. Consequently, the fatty acid composition of sea buckthorn products likely contributes to the developmental and viability effects observed in *Drosophila melanogaster*, while also supporting the broader nutritional relevance of these matrices.

Amino acids are essential regulators of growth, metabolism, and developmental timing in *Drosophila melanogaster* [83]. In the present study, sea buckthorn products contained significant amounts of amino acids, including all essential amino acids, mainly in seed flour and fruit pomace powder. In *Drosophila*, amino acids act as key nutritional signals that regulate larval growth through nutrient-sensing pathways, particularly the target of rapamycin (TOR) and insulin-like peptide (DILP) signaling, acting via the fat body and the brain [83,84]. Branched-chain amino acids, such as leucine and valine, are especially important activators of TOR signaling and are known to promote growth and developmental progression [42,85]. Therefore, the presence of these amino acids in fruit pomace powder may contribute to the improved viability and altered developmental dynamics observed under certain dietary conditions. Methionine plays a particularly significant role in *Drosophila* physiology. Previous studies have shown that restriction of methionine extends lifespan, while its supplementation accelerates development and increases fecundity, mainly through TOR activation [86,87]. Glutamine and proline are the main amino acid compounds in the protein fractions of sea buckthorn products. Using these, the metabolism of *Drosophila* produces α-ketoglutarate via glutamate intermediates, which enters the TCA (tricarboxylic acid) cycle and influences energy balance [88]. This may contribute to the accelerated developmental dynamics observed with the use of fruit pomace powder.

The free amino acid composition of *Drosophila* also varies across developmental stages, reflecting changing metabolic demands during larval growth, pupation, and adulthood [89]. Consequently, differences in amino acid availability provided by the sea buckthorn products may differentially affect larval growth, pupal development, and adult emergence, depending on dietary context, by modulating the nutrient-sensing and growth-regulatory pathways.

Sea buckthorn contains a diverse range of bioactive compounds, including water-soluble vitamin C, lipophilic tocopherols, polyphenols, flavonoids, and condensed tannins. In our study, seed flour showed the highest total polyphenol and flavonoid contents, followed by fruit pomace powder, whereas pulp and oil contained considerably lower amounts. Major phenolic acids identified included gallic acid and p-coumaric acid, while key flavonoids were naringenin, quercetin, kaempferol, isorhamnetin, and their derivatives. Condensed tannins, particularly prodelphinidin and catechin derivatives, were the most abundant in seed flour. Overall, the composition of these bioactive compounds in our samples aligns with previously reported profiles for sea buckthorn [10,90,91]. Flavonols, such as quercetin, kaempferol, and isorhamnetin, possess exceptional antioxidant, anti-inflammatory, anti-diabetic, and anticancer properties, making them invaluable in maintaining homeostasis in the body [92,93,94]. Rutin (quercetin-3-O-glycoside), mainly present in pulp and fruit pomace powder, acts as a potent antioxidant and cardiovascular protector, while also modulating inflammatory responses and metabolic pathways [95,96]. In flies, rutin may complement other flavonoids, promoting developmental stability and resilience under dietary stress.

Naringenin, a flavanone predominantly found in pulp and fruit pomace powder, has been shown to exhibit hepatoprotective, anti-atherogenic, anti-inflammatory, anti-mutagenic, and anticancer properties, and may enhance mitochondrial function and antioxidant defenses [97,98]. In *Drosophila*, these effects could stabilize cellular redox balance and support energy metabolism, potentially explaining the improved viability of larvae under high-sugar or low-nutrient conditions.

Condensed tannins, particularly prodelphinidin in seed flour and procyanidin in fruit pomace powder, may further enhance antioxidant capacity and influence nutrient utilization [99,100,101]. These compounds could contribute to the concentration-dependent effects observed for the different sea buckthorn products, supporting larval growth and adult emergence, especially under nutritional or metabolic stress. However, a high intake of tannins can have antinutritive effects and may increase indigestibility [102], which can contribute to the side effects associated with seed flour.

Although much of the research on these bioactive compounds has focused on human health—including cardiovascular, metabolic, and neuroprotective effects [10,19,69,91,92,95,96,97,98,99,100,101,103,104,105,106,107,108,109,110,111,112]—their fundamental antioxidant and metabolic regulatory functions are likely conserved across species.

The distinct composition of sea buckthorn matrices—including proteins, essential amino acids, fatty acids, polyphenols, flavonoids, and condensed tannins—likely underlies the product-specific effects on *Drosophila* development and viability, highlighting the combined role of macronutrients and bioactive compounds in shaping biological responses.

### 4.5. Integrated Summary of Compositional Features and Biological Effects in Drosophila melanogaster

To facilitate comparison between compositional features and biological outcomes, an integrated summary of the major amino acids, fatty acids, and observed effects in *Drosophila melanogaster* is presented in Table 5.

Overall, taking all parameters into account, fruit pomace powder proved to be the most effective sea buckthorn product among the tested products. It had a balanced fatty acid profile, with a favorable omega-3 to omega-6 fatty acid ratio and an appropriate distribution of saturated and monounsaturated fatty acids. In addition, it contained relatively high levels of several amino acids, such as asparagine, proline, and glutamine, while all essential amino acids were present at lower levels. The product was also characterized by significant amounts of bioactive compounds, which likely contributed to its antioxidant capacity.

Under a zero-nutrient diet, fruit pomace powder most effectively supported viability and, at higher concentrations, resulted in a growth curve remarkably similar to that observed with a normal-nutrient diet. Under normal-nutritional conditions, it moderately increased viability and slightly accelerated development, while under high-sugar conditions, viability remained at a level similar to the control and was accompanied by a slight acceleration of development. Taken together, these results suggest that fruit pomace powder provides a combination of nutrients and bioactive compounds that support development under different nutritional conditions without causing adverse effects.

## 5. Conclusions

This study provides a comprehensive characterization of four sea buckthorn products, demonstrating that their biological impact is strictly determined by their specific biochemical profiles. Our findings reveal a clear distinction between the fractions: while seed flour and fruit pomace powder are characterized by high protein and antioxidant levels, the seed oil offers a different matrix dominated by specific fatty acid profiles.

These differences directly translate into varying developmental effects. Specifically, the fruit pomace powder proved to be the most effective fraction, as its unique combination of nutrients significantly boosted developmental resilience in *Drosophila melanogaster*. This fraction supported normal growth even under extreme conditions, such as nutrient deficiency or high-sugar stress, suggesting that the synergy between macronutrients and bioactive compounds is key to the “superfood” status of the fruit.

Overall, these findings underscore that the biological potential of sea buckthorn products depends on their specific composition and that *Drosophila melanogaster* serves as a sensitive model to detect such effects. This work provides a foundation for future mechanistic studies on the interactions between nutrients, bioactive compounds, and organismal development, and may guide the optimization of sea buckthorn-based formulations for nutritional and functional applications. Furthermore, our research contributed to a comparative mapping of the nutritional value and bioactive effects of different parts of sea buckthorn berries, bringing us closer to demonstrating why this fruit is considered a true superfood.

While *Drosophila melanogaster* proved to be a sensitive model for detecting the biological effects of sea buckthorn products, extrapolation to humans or other organisms should be done with caution. This study tested only selected concentrations and specific dietary contexts and did not directly assess the bioavailability or metabolism of the individual compounds. Moreover, while correlations between nutrient and bioactive composition and developmental effects were observed, the precise mechanistic pathways remain to be elucidated. Future studies integrating additional models and mechanistic analyses would strengthen the understanding of the biological potential of sea buckthorn.

## Figures and Tables

**Table 1:** The composition of the used media.

Ingredients	Zero-Nutrient Medium	Normal-Nutrient Medium	High-Sugar Medium
Water (mL)	1000	1000	1000
Yeast paste (g)	-	70	70
Sucrose (g)	-	51.34 (0.15 M)	256.72 (0.75 M)
Wheat semolina (g)	-	30	30
Agar (g)	10	10	10
Nipagin E (g)	1	1	1

Note: M—molar.

**Table 2:** Quantitative fatty acid analysis of sea buckthorn matrices and their respective lipid fractions.

Type	Name	Product (g/100 g Product)				Fat Fraction (g/100 g Fat Fraction)			
		Seed Flour	Seed Oil	Pulp	Fruit Pomace Powder	Seed Flour	Seed Oil	Pulp	Fruit Pomace Powder
Saturated fatty acids (SFA)	Caprylic acid (C8:0)	<0.01	<0.01	<0.01	<0.01	0.03	<0.01	0.03	<0.01
	Capric acid (C10:0)	<0.01	<0.01	<0.01	<0.01	<0.01	<0.01	0.02	<0.01
	Lauric acid (C12:0)	<0.01	0.01	<0.01	0.01	0.06	0.01	0.12	0.07
	Myristic acid (C14:0)	0.01	0.18	0.02	0.07	0.25	0.18	0.60	0.65
	Pentadecanoic acid (C15:0)	0.01	0.14	0.01	0.02	0.21	0.14	0.19	0.16
	Palmitic acid (C16:0)	0.37	7.32	1.14	2.93	8.79	7.33	30.35	25.94
	Heptadecanoic acid (C17:0)	<0.01	0.05	<0.01	0.01	0.10	0.05	0.13	0.11
	Stearic acid (C18:0)	0.14	2.62	0.05	0.16	3.34	2.62	1.20	1.43
	Behenic acid (C22:0)	0.01	0.08	0.01	0.08	0.21	0.08	0.30	0.74
	Lignoceric acid (C24:0)	<0.01	0.01	0.01	0.08	<0.01	0.01	0.14	0.71
**Total SFA**		**0.54**	**10.41**	**1.24**	**3.37**	**12.99**	**10.42**	**33.08**	**29.81**
Monounsaturated fatty acids (MUFA)	Myristoleic acid (C14:1)	<0.01	<0.01	<0.01	<0.01	0.02	<0.01	0.03	0.02
	Palmitoleic acid (C16:1n7)	0.02	0.65	0.87	2.44	0.41	0.65	23.26	21.59
	Oleic acid (C18:1n9c)	0.88	18.40	0.85	2.09	21.06	18.42	22.63	18.47
	Elaidic acid (C18:1n9t)	<0.01	0.01	0.01	0.21	<0.01	0.01	0.33	1.86
	Erucic acid (C22:1n9)	<0.01	0.03	0.03	0.38	<0.01	0.03	0.87	3.33
**Total MUFA**		**0.90**	**19.09**	**1.77**	**5.12**	**21.49**	**19.11**	**47.12**	**45.27**
Polyunsaturated fatty acids (PUFA)	α-Linolenic acid (C18:3n3)	0.87	33.20	0.12	1.40	20.77	33.23	3.27	12.37
	cis-Linoleic acid (C18:2n6c)	1.84	36.77	0.60	1.32	44.10	36.81	16.03	11.64
	γ-Linoleic acid (C18:3n6)	0.02	0.42	0.01	0.06	0.54	0.42	0.31	0.54
	Docosadienoic acid (C22:2)	<0.01	0.01	0.01	0.04	0.07	0.01	0.19	0.34
	DHA (C22:6n3)	<0.01	<0.01	<0.01	<0.01	0.03	<0.01	0.03	<0.01
**Total PUFA**		**2.73**	**70.40**	**0.75**	**2.81**	**65.51**	**70.47**	**19.83**	**24.89**

Notes: Values represent the absolute content of individual fatty acids. Product columns refer to the concentration in the whole sample (seed flour, seed oil, pulp, and fruit pomace powder), while Fat fraction columns refer to the concentration within the extracted lipids. All analyses were performed by an accredited laboratory using GC-FID (Varian Inc., Palo Alto, CA, USA); single-point determinations were provided according to standard certification protocols. Values below 0.01 are reported as <0.01. SFA: Saturated Fatty Acids; MUFA: Monounsaturated Fatty Acids; PUFA: Polyunsaturated Fatty Acids. The color intensity represents the concentration level: darker shades indicate higher fatty acid content within the respective matrix or fat fraction. Only fatty acids present at concentrations ≥0.01 g/100 g in at least one sample are shown in Table 2. The complete fatty acid profile, including trace components, is provided in Supplementary Table S2.

**Table 3:** Amino acid profile and quantitative distribution in sea buckthorn matrices: absolute content (g/100 g product) and protein-based composition (g/100 g protein fraction).

Type	Name	Product (g/100 g Product)			Protein Fraction (g/100 g Protein Fraction)		
		Seed Flour	Pulp	Fruit Pomace Powder	Seed Flour	Pulp	Fruit Pomace Powder
Essential amino acids (EAA)	Arginine	1.90	0.01	0.27	8.15	0.85	2.85
	Histidine	0.40	0.02	0.22	1.72	1.69	2.32
	Isoleucine	0.65	0.03	0.31	2.79	2.54	3.27
	Leucine	1.33	0.04	0.48	5.71	3.39	5.07
	Lysine	1.12	0.08	0.79	4.81	6.78	8.34
	Methionine	0.08	0.01	0.03	0.34	0.85	0.32
	Phenylalanine	0.58	0.03	0.26	2.49	2.54	2.75
	Threonine	0.59	0.03	0.34	2.53	2.54	3.59
	Tryptophan	1.24	0.08	0.82	5.32	6.78	8.66
	Valine	0.84	0.05	0.43	3.61	4.24	4.54
**Total EAA**		**8.73**	**0.38**	**3.95**	**37.47**	**32.20**	**41.71**
Non-essential amino acids (N-EAA)	Alanine	1.17	0.04	0.51	5.02	3.39	5.39
	Asparagine	2.77	0.40	1.89	11.89	33.90	19.96
	Cysteine	0.05	0.01	0.05	0.21	0.85	0.53
	Glutamine	5.84	0.11	1.53	25.06	9.32	16.16
	Glycine	0.91	0.02	0.44	3.91	1.69	4.65
	Proline	1.68	0.21	1.34	7.21	17.80	14.15
	Serine	1.29	0.04	0.48	5.54	3.39	5.07
	Tyrosine	0.24	0.01	0.15	1.03	0.85	1.58
**Total N-EAA**		**13.95**	**0.84**	**6.39**	**59.87**	**71.19**	**67.48**

Notes: Values represent the absolute amino acid content. Product: whole sample; Protein fraction: concentration relative to the total protein content. The blue color intensity represents the concentration level: darker shades indicate higher amino acid content within the respective matrix or protein fraction. All measurements were conducted by an accredited laboratory (single-point determinations). EAA: Essential Amino Acids; N-EAA: Non-essential Amino Acids.

**Table 4:** Summary of phytochemicals found in various sea buckthorn matrices.

Group	Name	SF	SO	P	FPP	t_R_	[M + H]^+^	[M − H]^−^	F 1	F 2	F 3	F 4	F 5
Hydroxy-carboxylic acid	Quinic acid	+		+	+	1.86		191.06	173.04	171.03	127.04	111.04	
	Citric acid	+		+	+	2.34		191.02	173.01	129.02	111.01	87.01	
	Malic acid	+	+	+	+	2.35		133.01	115	89.02	87.01	72.99	
Phenolic acid	Gallic acid (3,4,5-Trihydroxybenzoic acid) *	+		+	+	3.34		169.01	125.02	97.03	69.03		
	p-Coumaric acid *			+	+	18.08		163.04	119.05				
	trans-Sinapic acid			+	+	20.03		223.06	208.04	193.01	179.07		
Flavonoid	Gallocatechin *	+		+	+	5.77		305.07	261.08	219.07	167.03	137.02	
	Catechin *	+		+	+	13.65		289.07	245.08	203.07	151.04		
	Epigallocatechin *	+		+	+	13.82		305.07	261.08	219.06	167.03		
	Epicatechin *	+		+	+	17.21		289.07	245.08	203.07	151.04		
	Isorhamnetin-3,4′-O-diglucoside			+	+	18.97		639.16	477.1	476.1	315.05	313.04	
	Quercetin-O-hexoside-O-rhamnoside	+				21.25		609.1	463.09	446.08	301.03		
	Isorhamnetin-3-O-sophoroside			+		21.88		639.16	315.05	314.04	300.03		
	Clitorin (Kaempferol-3-O-(2G-rhamnosyl-rutinoside)	+				22.19		739.2	285.04	284.03	255.03		
	Kaempferol-O-hexoside-O-rhamnoside	+				22.8		593.15	447.09	430.09	285.04		
	Isorhamnetin-O-hexoside-O-rhamnoside	+		+	+	22.99		623.16	477.1	461.11	315.05		
	Isoquercitrin (Quercetin-3-O-glucoside) *			+	+	23.01		463.09	301.04	300.03	271.03	255.03	
	Rutin (Quercetin-3-O-rutinoside) *			+	+	23.09		609.15	301.04	300.03	271.03		
	Isorhamnetin-3-O-glucoside *			+	+	25.06		477.1	315.05	314.04	299.02		
	Syringetin-3-O-glucoside			+	+	25.12		507.11	345.06	344.05	329.03		
	Isorhamnetin-3-O-rutinoside (Narcissin)	+		+	+	25.33		623.16	315.05	314.04	300.03	299.02	
	Syringetin-3-O-rutinoside			+	+	25.56		653.17	345.06	344.05	329.03		
	Naringenin (4′,5,7-Trihydroxyflavanone) *			+	+	27.56		271.06	177.02	165.02	151		
Vitamine	Nicotinamide			+		2.37	123.06		106.03	96.04	80.05		
Indolamine	Serotonine	+			+	3.45	177.1		160.07	142.06	132.08		
Condensed tannin	Prodelphinidin B isomer 1	+				3.46		609.14	441.08	423.07	305.06		
	Prodelphinidin B isomer 2	+				6.07		609.12	441.08	423.07	305.06		
	Procyanidin B				+	11.4		577.13	451.1	425.09	407.07		

(+): the phytochemical is present in the product; (*): confirmed by standard; SF—seed flour; SO—seed oil; P—pulp; FPP—fruit pomace powder; t_R_—retention time; [M + H]^+^—protonated molecular ion; [M − H]^−^—deprotonated molecular ion; F (1–5)—fragments.

**Table 5:** Overview of major compositional characteristics and biological effects of sea buckthorn matrices in *Drosophila melanogaster*.

Product	Main Fatty Acids	Main Amino Acids	Main Bioactive Compounds	Diet	Viability	Developmental Dynamics
Seed flour	cis-linoleic acid α-linolenic acid oleic acid	glutamine asparagine arginine proline	catechin and its derivatives kaempferol and its derivatives prodelphinidin	0N-diet	++	Delay *
				NN-diet	↔	No change
				HS-diet	↓/↔	No change
Seed oil	cis-linoleic acid α-linolenic acid oleic acid palmitic acid stearic acid	x	x	0N-diet	-	-
				NN-diet	↓	Acceleration
				HS-diet	↓/↔	Acceleration
Pulp	palmitic acid palmitoleic acid oleic acid cis-linoleic acid	asparagine glutamine proline	catechin and its derivatives isorhamnetin and its derivatives rutin naringenin	0N-diet	+	Delay *
				NN-diet	↑	Delay
				HS-diet	↑	Delay
Fruit pomace powder	palmitic acid palmitoleic acid oleic acid cis-linoleic acid α-linolenic acid	asparagine glutamine proline	catechin and its derivatives isorhamnetin and its derivatives rutin naringenin procyanidin	0N-diet	+++	No change/delay *
				NN-diet	↑	Acceleration
				HS-diet	↓/↔	Acceleration

x: no components; +++ create the highest viability between the products; ++ create the second highest viability between the products; + create the third highest viability between the products; - no viability; ↑ increase compared to control; ↓ decrease compared to control; ↔ no significant change. Developmental acceleration or delay refers to shifts in the developmental curve relative to the control group. * compared to the normal-nutrient diet control.

## Data Availability

The data presented in this study are available on request from the corresponding authors due to project-specific confidentiality and ongoing food product development.

## Supplementary Materials

The following supporting information can be downloaded at: https://www.mdpi.com/article/10.3390/nu18050824/s1, Table S1: The chemicals used for the chemical analyses. Table S2: Quantitative fatty acid analysis of sea buckthorn matrices and their respective lipid fractions.

## Abbreviations

The following abbreviations are used in this manuscript:

0N-diet	Zero-nutrient diet
AAE	Ascorbic acid equivalent
ABTS	2,2′-azino-bis(3-ethylbenzothiazoline-6-sulfonic acid)
CE	Catechin equivalent
CTC	Condensed tannin content
CVD	Cardiovascular disease
DPPH	2,2-diphenyl-1-picrylhydrazyl
EAA	Essential amino acid
FPP	Fruit pomace powder
FRAP	Ferric reducing antioxidant power
FW	Fresh weight
HS-diet	High-sugar diet
M	Molar
MUFA	Monounsaturated fatty acids
N	Normal
NN-diet	Normal-nutrient diet
N-EAA	Non-essential amino acids
P	Pulp
PA	Palmitic acid
PUFA	Polyunsaturated fatty acids
SB	Sea buckthorn
SF	Seed flour
SFA	Saturated fatty acids
SO	Seed oil
T2DM	Type 2 diabetes mellitus
TE	Trolox equivalent
TEAC	Trolox equivalent antioxidant capacity
TFC	Total flavonoid content
TPC	Total phenolic content
TPTZ	tri(2-pyridinyl)-1,3,5-triazine

## References

[1] Yuan H., Ma Q., Ye L., Piao G. (2016). The Traditional Medicine and Modern Medicine from Natural Products. Molecules.

[2] Li F., Wang Y., Li D., Chen Y., Dou Q.P. (2019). Are We Seeing a Resurgence in the Use of Natural Products for New Drug Discovery?. Expert Opin. Drug Discov..

[3] Li F.-S., Weng J.-K. (2017). Demystifying Traditional Herbal Medicine with Modern Approach. Nat. Plants.

[4] Keita K., Darkoh C., Okafor F. (2022). Secondary Plant Metabolites as Potent Drug Candidates against Antimicrobial-Resistant Pathogens. SN Appl. Sci..

[5] Pulingam T., Parumasivam T., Gazzali A.M., Sulaiman A.M., Chee J.Y., Lakshmanan M., Chin C.F., Sudesh K. (2022). Antimicrobial Resistance: Prevalence, Economic Burden, Mechanisms of Resistance and Strategies to Overcome. Eur. J. Pharm. Sci..

[6] Nwonu C., Ilesanmi O., Agbedahunsi J., Nwonu P. (2019). Natural Products as Veritable Source of Novel Drugs and Medicines: A Review. Int. J. Herb. Med..

[7] Gâtlan A.-M., Gutt G. (2021). Sea Buckthorn in Plant Based Diets. An Analytical Approach of Sea Buckthorn Fruits Composition: Nutritional Value, Applications, and Health Benefits. Int. J. Environ. Res. Public Health.

[8] Liu S., Xiao P., Kuang Y., Hao J., Huang T., Liu E. (2021). Flavonoids from Sea Buckthorn: A Review on Phytochemistry, Pharmacokinetics and Role in Metabolic Diseases. J. Food Biochem..

[9] Chen Y., Cai Y., Wang K., Wang Y. (2023). Bioactive Compounds in Sea Buckthorn and Their Efficacy in Preventing and Treating Metabolic Syndrome. Foods.

[10] Wang K., Xu Z., Liao X. (2021). Bioactive Compounds, Health Benefits and Functional Food Products of Sea Buckthorn: A Review. Crit. Rev. Food Sci. Nutr..

[11] Wang Z., Zhao F., Wei P., Chai X., Hou G., Meng Q. (2022). Phytochemistry, Health Benefits, and Food Applications of Sea Buckthorn (*Hippophae rhamnoides* L.): A Comprehensive Review. Front. Nutr..

[12] Wani T.A., Wani S.M., Ahmad M., Ahmad M., Gani A., Masoodi F.A. (2016). Bioactive Profile, Health Benefits and Safety Evaluation of Sea Buckthorn (*Hippophae rhamnoides* L.): A Review. Cogent Food Agric..

[13] Gutierres D., Pacheco R., Reis C.P. (2025). The Role of Omega-3 and Omega-6 Polyunsaturated Fatty Acid Supplementation in Human Health. Foods.

[14] Gao S., Guo Q., Qin C., Shang R., Zhang Z. (2017). Sea Buckthorn Fruit Oil Extract Alleviates Insulin Resistance through the PI3K/Akt Signaling Pathway in Type 2 Diabetes Mellitus Cells and Rats. J. Agric. Food Chem..

[15] Wu J., Shi S., Wang H., Wang S. (2016). Mechanisms Underlying the Effect of Polysaccharides in the Treatment of Type 2 Diabetes: A Review. Carbohydr. Polym..

[16] Bermúdez M.A., Pereira L., Fraile C., Valerio L., Balboa M.A., Balsinde J. (2022). Roles of Palmitoleic Acid and Its Positional Isomers, Hypogeic and Sapienic Acids, in Inflammation, Metabolic Diseases and Cancer. Cells.

[17] Dupak R., Hrnkova J., Simonova N., Kovac J., Ivanisova E., Kalafova A., Schneidgenova M., Prnova M.S., Brindza J., Tokarova K. (2022). The Consumption of Sea Buckthorn (*Hippophae rhamnoides* L.) Effectively Alleviates Type 2 Diabetes Symptoms in Spontaneous Diabetic Rats. Res. Vet. Sci..

[18] Chandra S., Zafar R., Dwivedi P., Shinde L., Prita B. (2018). Pharmacological and Nutritional Importance of Sea Buckthorn (Hippophae). Pharma Innov. J..

[19] Olas B. (2018). The Beneficial Health Aspects of Sea Buckthorn (*Elaeagnus rhamnoides* (L.) A.Nelson) Oil. J. Ethnopharmacol..

[20] Kopčeková J., Mrázová J., Fatrcová-Šramková K., Habánová M., Gažarová M., Lenártová P. (2023). Benefits of Sea Buckthorn Juice Consumption in Women of Productive Age with Hypercholesterolemia. Rocz. Państwowego Zakładu Hig..

[21] Murariu O.C., Lipșa F.D., Ulea E., Murariu F., Ciobanu M.-M., Frunză G., Cârlescu P.M., Stoica F., Diaconu N., Caruso G. (2025). Influence of Sea Buckthorn Fruit Part on Physical, Quality and Bioactive Properties of White Chocolate Under the Circular Economic Framework. Horticulturae.

[22] Murariu O.C., Lipșa F.D., Cârlescu P.M., Frunză G., Ciobanu M.M., Cara I.G., Murariu F., Stoica F., Albu A., Tallarita A.V. (2024). The Effect of Including Sea Buckthorn Berry By-Products on White Chocolate Quality and Bioactive Characteristics under a Circular Economy Context. Plants.

[23] Eickelberg V., Lüersen K., Staats S., Rimbach G. (2022). Phenotyping of *Drosophila melanogaster*—A Nutritional Perspective. Biomolecules.

[24] Álvarez-Rendón J.P., Salceda R., Riesgo-Escovar J.R. (2018). *Drosophila melanogaster* as a Model for Diabetes Type 2 Progression. BioMed Res. Int..

[25] (1980). Tartósított Élelmiszerek Nedvességtartalmának Meghatározása (Determination of Moisture Content in Preserved Foods).

[26] (1987). Élelmiszerek és Élvezeti Cikkek Nitrogéntartalmának Meghatározása Kjeldahl-Féle Módszerrel (Determination of Nitrogen Content in Foodstuffs and Luxury Foods Using the Kjeldahl Method).

[27] (1985). Tartósított Élelmiszerek Zsírtartalmának Meghatározása (Determination of Fat Content in Preserved Foods).

[28] (2008). Codex Alimentarius Hungaricus (Hungarian Food Book).

[29] (1986). Tartósított Élelmiszerek Cukortartalmának Meghatározása (Determination of Sugar Content in Preserved Foods).

[30] Lásztity R., Dezső T. (1987). Élelmiszer Analitika Elméleti Alapjai I.

[31] Spies J.R., Chambers D.C. (1950). Determination of Tryptophan with P-Dimethylaminobenzaldehyde Using Photochemical Development of Color. Anal. Chem..

[32] Neamtu A.-A., Szoke-Kovacs R., Mihok E., Georgescu C., Turcus V., Olah N.K., Frum A., Tita O., Neamtu C., Szoke-Kovacs Z. (2020). Bilberry (*Vaccinium myrtillus* L.) Extracts Comparative Analysis Regarding Their Phytonutrient Profiles, Antioxidant Capacity along with the In Vivo Rescue Effects Tested on a *Drosophila melanogaster* High-Sugar Diet Model. Antioxidants.

[33] Benzie I.F.F., Strain J.J. (1996). The Ferric Reducing Ability of Plasma (FRAP) as a Measure of “Antioxidant Power”: The FRAP Assay. Anal. Biochem..

[34] Re R., Pellegrini N., Proteggente A., Pannala A., Yang M., Rice-Evans C. (1999). Antioxidant Activity Applying an Improved ABTS Radical Cation Decolorization Assay. Free Radic. Biol. Med..

[35] Blois M.S. (1958). Antioxidant Determinations by the Use of a Stable Free Radical. Nature.

[36] Singleton V.L., Rossi J.A. (1965). Colorimetry of Total Phenolics with Phosphomolybdic-Phosphotungstic Acid Reagents. Am. J. Enol. Vitic..

[37] Zhishen J., Mengcheng T., Jianming W. (1999). The Determination of Flavonoid Contents in Mulberry and Their Scavenging Effects on Superoxide Radicals. Food Chem..

[38] Price M.L., Van Scoyoc S., Butler L.G. (1978). A Critical Evaluation of the Vanillin Reaction as an Assay for Tannin in Sorghum Grain. J. Agric. Food Chem..

[39] Hagerman A.E. (2002). Vanillin Assay. https://www.scribd.com/document/326357563/Vanillin-Assay.

[40] Venn B.J. (2020). Macronutrients and Human Health for the 21st Century. Nutrients.

[41] Banik S., Hossain M. (2014). A Comparative Overview on Good Fats and Bad Fats: Guide to Control Healthy Body. Int. J. Health Sci..

[42] Khadka Y.R. (2021). Amino Acid- Essentiality to Human Body. Patan Pragya.

[43] Anas M., Solanki T.V., Bakal R.L., Hatwar P.R. (2025). Phytochemicals: A Review of Their Role in Human Health and Disease Prevention. GSC Biol. Pharm. Sci..

[44] Munteanu I.G., Apetrei C. (2021). Analytical Methods Used in Determining Antioxidant Activity: A Review. Int. J. Mol. Sci..

[45] Rudrapal M., Khairnar S.J., Khan J., Dukhyil A.B., Ansari M.A., Alomary M.N., Alshabrmi F.M., Palai S., Deb P.K., Devi R. (2022). Dietary Polyphenols and Their Role in Oxidative Stress-Induced Human Diseases: Insights Into Protective Effects, Antioxidant Potentials and Mechanism(s) of Action. Front. Pharmacol..

[46] Van Dam E., Van Leeuwen L.A.G., Dos Santos E., James J., Best L., Lennicke C., Vincent A.J., Marinos G., Foley A., Buricova M. (2020). Sugar-Induced Obesity and Insulin Resistance Are Uncoupled from Shortened Survival in Drosophila. Cell Metab..

[47] Vatashchuk M.V., Bayliak M.M., Hurza V.V., Storey K.B., Lushchak V.I. (2022). Metabolic Syndrome: Lessons from Rodent and *Drosophila* Models. BioMed Res. Int..

[48] Etuh M.A., Ohemu L.T., Pam D.D. (2021). *Lantana camara* Ethanolic Leaves Extracts Exhibit Anti-Aging Properties in *Drosophila melanogaster*: Survival-Rate and Life Span Studies. Toxicol. Res..

[49] Sang J., Dhakal S., Lee Y. (2021). Cucurbitacin B Suppresses Hyperglycemia Associated with a High Sugar Diet and Promotes Sleep in *Drosophila melanogaster*. Mol. Cells.

[50] Wang M., Mao H., Chen J., Qi L., Wang J. (2022). Ameliorative Effect of Bayberry Leaves Proanthocyanidins on High Sugar Diet Induced *Drosophila melanogaster*. Front. Pharmacol..

[51] Palanker Musselman L., Fink J.L., Narzinski K., Ramachandran P.V., Sukumar Hathiramani S., Cagan R.L., Baranski T.J. (2011). A High-Sugar Diet Produces Obesity and Insulin Resistance in Wild-Type *Drosophila*. Dis. Models Mech..

[52] Radenkovs V., Püssa T., Juhnevica-Radenkova K., Anton D., Seglina D. (2018). Phytochemical Characterization and Antimicrobial Evaluation of Young Leaf/Shoot and Press Cake Extracts from *Hippophae rhamnoides* L.. Food Biosci..

[53] Héjja M., György É., Lóga F.Á., Nagy R., Pacza T., Sipos P., Tankó G., Laslo É., Mészáros N., Turcuș V. (2025). Gemmotherapy Extracts Like the Dog Rose, Lingonberry, Sea Buckthorn, Blackthorn, Common Grape, Hawthorn, Raspberry and Boxwood Feature Variable Yet Excelling Antimicrobial Effects. Antibiotics.

[54] Bal L.M., Meda V., Naik S.N., Satya S. (2011). Sea Buckthorn Berries: A Potential Source of Valuable Nutrients for Nutraceuticals and Cosmoceuticals. Food Res. Int..

[55] Nour V., Panaite T.D., Corbu A.R., Ropota M., Turcu R.P. (2021). Nutritional and Bioactive Compounds in Dried Sea-Buckthorn Pomace. Erwerbs-Obstbau.

[56] Máté M., Selimaj G., Simon G., Szalóki-Dorkó L., Ficzek G. (2022). Assessment of Berries of Some Sea Buckthorn Genotypes by Physicochemical Properties and Fatty Acid Content of the Seed. Plants.

[57] Hao W., He Z., Zhu H., Liu J., Kwek E., Zhao Y., Ma K.Y., He W.-S., Chen Z.-Y. (2019). Sea Buckthorn Seed Oil Reduces Blood Cholesterol and Modulates Gut Microbiota. Food Funct..

[58] Guo R., Chang X., Guo X., Brennan C.S., Li T., Fu X., Liu R.H. (2017). Phenolic Compounds, Antioxidant Activity, Antiproliferative Activity and Bioaccessibility of Sea Buckthorn (*Hippophaë rhamnoides* L.) Berries as Affected by in Vitro Digestion. Food Funct..

[59] Ivanišová E., Mittuchová K., Mareček J., Frančáková H., Klymenko S. (2017). Small Berries—Attractive Source Of Bioactive Compounds For Consumers. Agrobiodiversity for Improving Nutrition, Health and Life Quality.

[60] McKechnie S.W., Geer B.W. (1993). Long-Chain Dietary Fatty Acids Affect the Capacity of *Drosophila melanogaster* to Tolerate Ethanol. J. Nutr..

[61] Carta G., Murru E., Banni S., Manca C. (2017). Palmitic Acid: Physiological Role, Metabolism and Nutritional Implications. Front. Physiol..

[62] Korbecki J., Bajdak-Rusinek K. (2019). The Effect of Palmitic Acid on Inflammatory Response in Macrophages: An Overview of Molecular Mechanisms. Inflamm. Res..

[63] Wang X., Zhang C., Bao N. (2023). Molecular Mechanism of Palmitic Acid and Its Derivatives in Tumor Progression. Front. Oncol..

[64] Annevelink C.E., Sapp P.A., Petersen K.S., Shearer G.C., Kris-Etherton P.M. (2023). Diet-Derived and Diet-Related Endogenously Produced Palmitic Acid: Effects on Metabolic Regulation and Cardiovascular Disease Risk. J. Clin. Lipidol..

[65] Mthembu S.X.H., Mazibuko-Mbeje S.E., Silvestri S., Orlando P., Marcheggiani F., Cirilli I., Nkambule B.B., Muller C.J.F., Tiano L., Dludla P.V. (2024). Low Levels and Partial Exposure to Palmitic Acid Improves Mitochondrial Function and the Oxidative Status of Cultured Cardiomyoblasts. Toxicol. Rep..

[66] Coniglio S., Shumskaya M., Vassiliou E. (2023). Unsaturated Fatty Acids and Their Immunomodulatory Properties. Biology.

[67] Zárate R., El Jaber-Vazdekis N., Tejera N., Pérez J.A., Rodríguez C. (2017). Significance of Long Chain Polyunsaturated Fatty Acids in Human Health. Clin. Transl. Med..

[68] Los D.A., Murata N. (2004). Membrane Fluidity and Its Roles in the Perception of Environmental Signals. Biochim. Biophys. Acta (BBA)—Biomembr..

[69] Oppedisano F., Macrì R., Gliozzi M., Musolino V., Carresi C., Maiuolo J., Bosco F., Nucera S., Caterina Zito M., Guarnieri L. (2020). The Anti-Inflammatory and Antioxidant Properties of n-3 PUFAs: Their Role in Cardiovascular Protection. Biomedicines.

[70] Oliver L., Dietrich T., Marañón I., Villarán M.C., Barrio R.J. (2020). Producing Omega-3 Polyunsaturated Fatty Acids: A Review of Sustainable Sources and Future Trends for the EPA and DHA Market. Resources.

[71] Dyall S.C. (2015). Long-Chain Omega-3 Fatty Acids and the Brain: A Review of the Independent and Shared Effects of EPA, DPA and DHA. Front. Aging Neurosci..

[72] Ji A., Diao H., Wang X., Yang R., Zhang J., Luo W., Cao R., Cao Z., Wang F., Cai T. (2012). N-3 Polyunsaturated Fatty Acids Inhibit Lipopolysaccharide-Induced Microglial Activation and Dopaminergic Injury in Rats. NeuroToxicology.

[73] Choi J., Jang J., Son D., Im H.-S., Kim J., Park J., Choi W., Han S.-B., Hong J. (2017). Antarctic Krill Oil Diet Protects against Lipopolysaccharide-Induced Oxidative Stress, Neuroinflammation and Cognitive Impairment. Int. J. Mol. Sci..

[74] Simopoulos A.P., DiNicolantonio J.J. (2016). The Importance of a Balanced ω-6 to ω-3 Ratio in the Prevention and Management of Obesity. Open Heart.

[75] Beaulieu J., Costa G., Renaud J., Moitié A., Glémet H., Sergi D., Martinoli M.-G. (2021). The Neuroinflammatory and Neurotoxic Potential of Palmitic Acid Is Mitigated by Oleic Acid in Microglial Cells and Microglial-Neuronal Co-Cultures. Mol. Neurobiol..

[76] Frigolet M.E., Gutiérrez-Aguilar R. (2017). The Role of the Novel Lipokine Palmitoleic Acid in Health and Disease. Adv. Nutr..

[77] Yang Z.-H., Miyahara H., Hatanaka A. (2011). Chronic Administration of Palmitoleic Acid Reduces Insulin Resistance and Hepatic Lipid Accumulation in KK-Ay Mice with Genetic Type 2 Diabetes. Lipids Health Dis..

[78] Astrup A., Magkos F., Bier D.M., Brenna J.T., De Oliveira Otto M.C., Hill J.O., King J.C., Mente A., Ordovas J.M., Volek J.S. (2020). Saturated Fats and Health: A Reassessment and Proposal for Food-Based Recommendations. J. Am. Coll. Cardiol..

[79] Lawrence G.D. (2024). Saturated Fats: Time to Assess Their Beneficial Role in a Healthful Diet. Dietetics.

[80] Kang Z.-Q., Yang Y., Xiao B. (2020). Dietary Saturated Fat Intake and Risk of Stroke: Systematic Review and Dose–Response Meta-Analysis of Prospective Cohort Studies. Nutr. Metab. Cardiovasc. Dis..

[81] Simopoulos A.P. (2002). The Importance of the Ratio of Omega-6/Omega-3 Essential Fatty Acids. Biomed. Pharmacother..

[82] Akerele O.A., Cheema S.K. (2016). A Balance of Omega-3 and Omega-6 Polyunsaturated Fatty Acids Is Important in Pregnancy. J. Nutr. Intermed. Metab..

[83] Oi A., Obata F. (2025). Nutrient Sensing and Signalling of Specific Amino Acids: Insights from Drosophila Study. Curr. Opin. Cell Biol..

[84] Manière G., Alves G., Berthelot-Grosjean M., Grosjean Y. (2020). Growth Regulation by Amino Acid Transporters in Drosophila Larvae. Cell. Mol. Life Sci..

[85] Martelli F., Quig A., Mele S., Lin J., Fulton T.L., Wansbrough M., Barlow C.K., Schittenhelm R.B., Johnson T.K., Piper M.D.W. (2024). A Defined Diet for Pre-Adult *Drosophila melanogaster*. Sci. Rep..

[86] Grandison R.C., Piper M.D.W., Partridge L. (2009). Amino-Acid Imbalance Explains Extension of Lifespan by Dietary Restriction in Drosophila. Nature.

[87] Hoedjes K.M., Rodrigues M.A., Flatt T. (2017). Amino Acid Modulation of Lifespan and Reproduction in Drosophila. Curr. Opin. Insect Sci..

[88] Cox J.E., Thummel C.S., Tennessen J.M. (2017). Metabolomic Studies in *Drosophila*. Genetics.

[89] Sivakumar S.P., Mohanraj R.S., Saminathan P. (2025). Comparative Analysis of Free Amino Acids in Eggs, Larvae, and Adults of *Drosophila melanogaster*. Int. J. Entomol. Res..

[90] Teleszko M., Wojdyło A., Rudzińska M., Oszmiański J., Golis T. (2015). Analysis of Lipophilic and Hydrophilic Bioactive Compounds Content in Sea Buckthorn (*Hippophaë rhamnoides* L.) Berries. J. Agric. Food Chem..

[91] Dienaitė L., Pukalskas A., Pukalskienė M., Pereira C.V., Matias A.A., Venskutonis P.R. (2020). Phytochemical Composition, Antioxidant and Antiproliferative Activities of Defatted Sea Buckthorn (*Hippophaë rhamnoides* L.) Berry Pomace Fractions Consecutively Recovered by Pressurized Ethanol and Water. Antioxidants.

[92] Martiniakova M., Penzes N., Biro R., Sarocka A., Kovacova V., Mondockova V., Ciernikova S., Omelka R. (2024). Sea Buckthorn and Its Flavonoids Isorhamnetin, Quercetin, and Kaempferol Favorably Influence Bone and Breast Tissue Health. Front. Pharmacol..

[93] Aghababaei F., Hadidi M. (2023). Recent Advances in Potential Health Benefits of Quercetin. Pharmaceuticals.

[94] Yang L., Gao Y., Bajpai V.K., El-Kammar H.A., Simal-Gandara J., Cao H., Cheng K.-W., Wang M., Arroo R.R.J., Zou L. (2021). Advance toward Isolation, Extraction, Metabolism and Health Benefits of Kaempferol, a Major Dietary Flavonoid with Future Perspectives. Crit. Rev. Food Sci. Nutr..

[95] Semwal R., Joshi S.K., Semwal R.B., Semwal D.K. (2021). Health Benefits and Limitations of Rutin—A Natural Flavonoid with High Nutraceutical Value. Phytochem. Lett..

[96] Yong D.O.C., Saker S.R., Chellappan D.K., Madheswaran T., Panneerselvam J., Choudhury H., Pandey M., Chan Y.L., Collet T., Gupta G. (2020). Molecular and Immunological Mechanisms Underlying the Various Pharmacological Properties of the Potent Bioflavonoid, Rutin. Endocr. Metab. Immune Disord. Drug Targets.

[97] Salehi B., Fokou P.V.T., Sharifi-Rad M., Zucca P., Pezzani R., Martins N., Sharifi-Rad J. (2019). The Therapeutic Potential of Naringenin: A Review of Clinical Trials. Pharmaceuticals.

[98] Lai Z., Ke L., Zhao W. (2025). Naringenin as a Neurotherapeutic Agent in Alzheimer’s Disease: Epigenetic Signatures, Gut Microbiota Alterations, and Molecular Neuroprotection. Front. Aging Neurosci..

[99] Rauf A., Imran M., Abu-Izneid T., Iahtisham-Ul-Haq, Patel S., Pan X., Naz S., Sanches Silva A., Saeed F., Rasul Suleria H.A. (2019). Proanthocyanidins: A Comprehensive Review. Biomed. Pharmacother..

[100] Wang Y., Zhang L., Xiao H., Ye X., Pan H., Chen S. (2024). Revisiting Dietary Proanthocyanidins on Blood Glucose Homeostasis from a Multi-Scale Structural Perspective. Curr. Res. Food Sci..

[101] Lee Y. (2017). Cancer Chemopreventive Potential of Procyanidin. ToxicolRes.

[102] Hoque M.B., Tanjila M.J., Hosen M.I., Hannan M.A., Haque P., Rahman M.M., Hasan T. (2025). A Comprehensive Review of the Health Effects, Origins, Uses, and Safety of Tannins. Plant Soil.

[103] Yuan H., Huang H., Du Y., Zhao J., Yu S., Lin Y., Chen Y., Shan C., Zhao Y., Belwal T. (2025). Sea Buckthorn Polyphenols on Gastrointestinal Health and the Interactions with Gut Microbiota. Food Chem..

[104] Yu L., Chen C., Wang L.-F., Kuang X., Liu K., Zhang H., Du J.-R. (2013). Neuroprotective Effect of Kaempferol Glycosides against Brain Injury and Neuroinflammation by Inhibiting the Activation of NF-κB and STAT3 in Transient Focal Stroke. PLoS ONE.

[105] Wu P., Meng X., Zheng H., Zeng Q., Chen T., Wang W., Zhang X., Su J. (2018). Kaempferol Attenuates ROS-Induced Hemolysis and the Molecular Mechanism of Its Induction of Apoptosis on Bladder Cancer. Molecules.

[106] Oboh G., Ademiluyi A.O., Akinyemi A.J., Henle T., Saliu J.A., Schwarzenbolz U. (2012). Inhibitory Effect of Polyphenol-Rich Extracts of Jute Leaf (*Corchorus olitorius*) on Key Enzyme Linked to Type 2 Diabetes (α-Amylase and α-Glucosidase) and Hypertension (Angiotensin I Converting) in Vitro. J. Funct. Foods.

[107] Masoodi K.Z., Wani W., Dar Z.A., Mansoor S., Anam-ul-Haq S., Farooq I., Hussain K., Wani S.A., Nehvi F.A., Ahmed N. (2020). Sea Buckthorn (*Hippophae rhamnoides* L.) Inhibits Cellular Proliferation, Wound Healing and Decreases Expression of Prostate Specific Antigen in Prostate Cancer Cells in Vitro. J. Funct. Foods.

[108] Mihal M., Roychoudhury S., Sirotkin A.V., Kolesarova A. (2023). Sea Buckthorn, Its Bioactive Constituents, and Mechanism of Action: Potential Application in Female Reproduction. Front. Endocrinol..

[109] Olas B., Skalski B. (2022). Preparations from Various Organs of Sea Buckthorn (*Elaeagnus rhamnoides* (L.) A. Nelson) as Important Regulators of Hemostasis and Their Role in the Treatment and Prevention of Cardiovascular Diseases. Nutrients.

[110] Ganbold M., Owada Y., Ozawa Y., Shimamoto Y., Ferdousi F., Tominaga K., Zheng Y.-W., Ohkohchi N., Isoda H. (2019). Isorhamnetin Alleviates Steatosis and Fibrosis in Mice with Nonalcoholic Steatohepatitis. Sci. Rep..

[111] Afonina I.S., Zhong Z., Karin M., Beyaert R. (2017). Limiting Inflammation—The Negative Regulation of NF-κB and the NLRP3 Inflammasome. Nat. Immunol..

[112] Chen J., Zhong H., Huang Z., Chen X., You J., Zou T. (2023). A Critical Review of Kaempferol in Intestinal Health and Diseases. Antioxidants.

