# Supplementary material for: Chemical Profiling and In Vivo Evaluation of Sea Buckthorn-Derived Matrices in Drosophila melanogaster Under Varied Dietary Regimes
Source: Nutrients. 2026 Mar 3;18(5):824. doi: 10.3390/nu18050824 (PMC12987292; doi:10.3390/nu18050824)
Supplement: Supplementary file 1 [file nutrients-18-00824-s001.zip › nutrients-4166615-supplementary.pdf]

Table S1. The chemicals used for the chemical analyses

Name	Abbreviation/ Formula	Company	Method
2,2′-azino-bis(3-ethylbenzothiazoline-6-sulfonic acid)	ABTS	Merck, Darmstadt, Germany	• ABTS assay
2,2-diphenyl-1-picrylhydrazyl	DPPH	Merck, Darmstadt, Germany	• DPPH assay
4-hidroxi-3-metoxibenzaldehid	vanillin	Thermo Fisher, Kandel, Germany	• condensed tannin content
6-hydroxy-2,5,7,8-tetramethylchroman-2-carboxylic acid	Trolox	Thermo Fisher Scientific, Waltham, USA	• ABTS assay,<br>• DPPH assay
α-amilase	-	Megazyme International Ireland Limited, Bray, Ireland	• dietary fiber content
Acetic acid	CH_3COOH	VWR International, Leuven, Belgium	• FRAP assay,<br>• total sugar content
acetone	CH_3COCH_3	VWR International, Leuven, Belgium	• dietary fiber content
aluminium-chloride	$AlCl_3$	VWR International, Leuven, Belgium	• total flavonoid content
amyloglucosidase	-	Megazyme International Ireland Limited, Bray, Ireland	• dietary fiber content
Ascorbic acid	$C_6H_8O_6$	VWR International, Leuven, Belgium	• FRAP assay
boric acid	H_3BO_3	VWR International, Leuven, Belgium	• protein content
boron trifluoride-methanol solution	BF_3 : MeOH	Sigma Aldrich, Steinheim, Germany	• Fatty acid profile
Carrez I	-	VWR International, Les Aires, France	• total carbohydrate content,<br>• sodium chloride content
Carrez II	-	VWR International, Les Aires, France	• total carbohydrate content,<br>• sodium chloride content
catechin	$C_{15}H_{14}O_6$	Merck, Darmstadt, Germany	• total flavonoid content
Ethanol	CH_3CH_2OH	VWR International, Leuven, Belgium	• dietary fiber content
Formic acid	HCOOH	VWR International, Leuven, Belgium	• phytonutrient profile
gallic-acid	$C_7H_6O_5$	Thermo Fisher, Kandel, Germany	• total phenolic content
glucose	$C_6H_{12}O_6$	VWR International, Leuven, Belgium	• total carbohydrate content
hydrochloric acid	HCl	VWR International, Fon-tenay-sous-Bois-cedex, France	• FRAP assay,<br>• ABTS assay,<br>• condensed tannin content,<br>• total sugar content,<br>• dietary fiber content

iodine	I	VWR International, Leuven, Belgium	• total sugar content
Iron (III) chloride	$FeCl_3$	VWR International, Leuven, Belgium	• FRAP assay
Luff-solution	-	VWR International, Leuven, Belgium	• total sugar content
methanol	CH_3OH	VWR International, Rosny-sous-Bois, France	• DPPH assay
n-hexane	C_6H_{14}	VWR International, Leuven, Belgium	• fatty acid profile
petroleum ether	-	VWR International, Rue d'Carnot, Fonteney-sous-Bois, France	• total fat content
potassium chromate	K_2CrO_4	VWR International, Leuven, Belgium	• sodium chloride content
potassium persulfate	$K_2S_2O_8$	VWR International, Leuven, Belgium	• ABTS assay
protease	-	Megazyme International Ireland Limited, Bray, Ireland	• dietary fiber content
silver nitrate	$AgNO_3$	VWR International, Leuven, Belgium	• sodium chloride content
sodium 1,2-naphthoquinone-4-sulfonate	Folin reagent	VWR International, Leuven, Belgium	• total phenolic content
sodium carbonate	Na_2CO_3	VWR International, Leuven, Belgium	• total phenolic content
Sodium chloride	NaCl	VWR International, Leuven, Belgium	• fatty acid profile
sodium nitrite	$NaNO_2$	VWR International, Leuven, Belgium	• total phenolic content
Sodium sulphate	N_2SO_4	VWR International, Leuven, Belgium	• fatty acid profile
Sodium-acetate 3H_2O	$CH_3COONa{\cdot}3H_2O$	VWR International, Leuven, Belgium	• FRAP assay
sodium-hydroxide	NaOH	VWR International, Leuven, Belgium	• total flavonoid content,<br>• protein content,<br>• dietary fiber content
sodium-thiosulphate	$Na_2S_2O_3$	VWR International, Leuven, Belgium	• total sugar content
starch	$(C_6H_{10}O_5)_n$	VWR International, Leuven, Belgium	• total sugar content
sulphuric acid	H_2SO_4	VWR International, Leuven, Belgium	• protein content,<br>• total carbohydrate content
tri(2-pyridinyl)-1,3,5-triazine	TPTZ	Thermo Fisher Scientific, Waltham, USA	• FRAP assay

Table S2. Quantitative fatty acid analysis of sea buckthorn matrices and their respective lipid fractions.

Type	Name	Product (g/100 g product)				Fat fraction (g/100 g fat fraction)			
		Seed flour	Seed oil	Pulp	Fruit pomace powder	Seed flour	Seed oil	Pulp	Fruit pomace powder
SFA	Butyric acid (C4:0)	<0.01	<0.01	<0.01	<0.01	<0.01	<0.01	<0.01	<0.01
	Caproic acid (C6:0)	<0.01	<0.01	<0.01	<0.01	0.01	<0.01	0.01	0.01
	Caprylic acid (C8:0)	<0.01	<0.01	<0.01	<0.01	0.03	<0.01	0.03	<0.01
	Capric acid (C10:0)	<0.01	<0.01	<0.01	<0.01	<0.01	<0.01	0.02	<0.01
	Undecanoic acid (C11:0)	<0.01	<0.01	<0.01	<0.01	<0.01	<0.01	<0.01	<0.01
	Lauric acid (C12:0)	<0.01	0.01	<0.01	0.01	0.06	0.01	0.12	0.07
	Tridecanoic acid (C13:0)	<0.01	<0.01	<0.01	<0.01	<0.01	<0.01	<0.01	<0.01
	Myristic acid (C14:0)	0.01	0.18	0.02	0.07	0.25	0.18	0.6	0.65
	Pentadecanoic acid (C15:0)	0.01	0.14	0.01	0.02	0.21	0.14	0.19	0.16
	Palmitic acid (C16:0)	0.37	7.32	1.14	2.93	8.79	7.33	30.35	25.94
	Heptadecanoic acid (C17:0)	<0.01	0.05	0.00	0.01	0.1	0.05	0.13	0.11
	Stearic acid (C18:0)	0.14	2.62	0.05	0.16	3.34	2.62	1.2	1.43
	Arachidic acid (C20:0)	<0.01	<0.01	<0.01	<0.01	<0.01	<0.01	<0.01	<0.01
	Heneicosanoic acid (C21:0)	<0.01	<0.01	<0.01	<0.01	<0.01	<0.01	<0.01	<0.01
	Behenic acid (C22:0)	0.01	0.08	0.01	0.08	0.21	0.08	0.3	0.74
	Tricosanoic acid (C23:0)	<0.01	<0.01	<0.01	<0.01	<0.01	<0.01	<0.01	<0.01
	Lignoceric acid (C24:0)	<0.01	0.01	0.01	0.08	<0.01	0.01	0.14	0.71
MUFA	Myristoleic acid (C14:1)	<0.01	<0.01	<0.01	<0.01	0.02	0	0.03	0.02
	cis-10-Pentadecanoic acid (C15:1)	<0.01	<0.01	<0.01	<0.01	<0.01	<0.01	<0.01	<0.01
	cis-10-Heptadecanoic acid (C17:1)	<0.01	<0.01	<0.01	<0.01	<0.01	<0.01	<0.01	<0.01
	Elaidic acid (C18:1n9t)	<0.01	0.01	0.01	0.21	<0.01	0.01	0.33	1.86
	Oleic acid (C18:1n9c)	0.88	18.40	0.85	2.09	21.06	18.42	22.63	18.47
	cis-11-Eikosenoic acid (C20:1n9)	<0.01	<0.01	<0.01	<0.01	<0.01	<0.01	<0.01	<0.01
	Erucic acid (C22:1n9)	<0.01	0.03	0.03	0.38	<0.01	0.03	0.87	3.33
	Nervonic acid (C24:1n9)	<0.01	<0.01	<0.01	<0.01	0.01	0	0.01	0.01
	Palmitoleic acid (C16:1n7)	0.02	0.65	0.87	2.44	0.41	0.65	23.26	21.59
PUFA	trans-Linoleic acid (C18:2n6t)	<0.01	<0.01	<0.01	<0.01	<0.01	<0.01	<0.01	<0.01

cis-Linoleic acid (C18:2n6c)	1.84	36.77	0.60	1.32	44.1	36.81	16.03	11.64
γ-Linoleic acid (C18:3n6)	0.02	0.42	0.01	0.06	0.54	0.42	0.31	0.54
α-Linolenic acid (C18:3n3)	0.87	33.20	0.12	1.40	20.77	33.23	3.27	12.37
cis-11,14-Eikosadienoic acid (C20:2n6)	<0.01	<0.01	<0.01	<0.01	<0.01	<0.01	<0.01	<0.01
cis-8,11,14-Eikosatrienoic acid (C20:3n6)	<0.01	<0.01	<0.01	<0.01	<0.01	0.01	<0.01	<0.01
11,14,17-Eikosatrienoic acid (C20:3n3)	<0.01	<0.01	<0.01	<0.01	<0.01	<0.01	<0.01	<0.01
Arachidonic acid (C20:4n6)	<0.01	<0.01	<0.01	<0.01	<0.01	<0.01	<0.01	<0.01
cis-13,16-Docosadienoic acid (C22:2)	<0.01	0.01	0.01	0.04	0.07	0.01	0.19	0.34
cis-5,8,11,14,17-Eicosapentaenoic acid (C20:5n3)	<0.01	<0.01	<0.01	<0.01	<0.01	<0.01	<0.01	<0.01
cis-4,7,10,13,16,19-Docosahexaenoic acid (C22:6n3)	<0.01	<0.01	<0.01	<0.01	0.03	<0.01	0.03	<0.01
